# Nanosheets of a Layered Metal–Organic Framework for Separation of CO_2_/CH_4_ using Mixed Matrix Membranes

**DOI:** 10.1021/acsami.4c05611

**Published:** 2024-06-13

**Authors:** Meng He, Yinlin Chen, Wanpeng Lu, Lixia Guo, Kui Hu, Xue Han, Inigo Vitorica-Yrezabal, Catherine Dejoie, Andrew N. Fitch, Martin Schröder, Sihai Yang

**Affiliations:** † Department of Chemistry, University of Manchester, Manchester M13 9PL, U.K.; ‡ College of Chemistry and Molecular Engineering, Beijing National Laboratory for Molecular Sciences, Peking University, Beijing 100871, China; § College of Chemistry, Beijing Normal University, Beijing 100875, China; ∥ The European Synchrotron Radiation Facility, 71 Avenue des Martyrs CS40220 Grenoble Cedex 9 38043, France

**Keywords:** metal−organic framework, nanosheet, powder diffraction, mixed-matrix membranes, gas separation

## Abstract

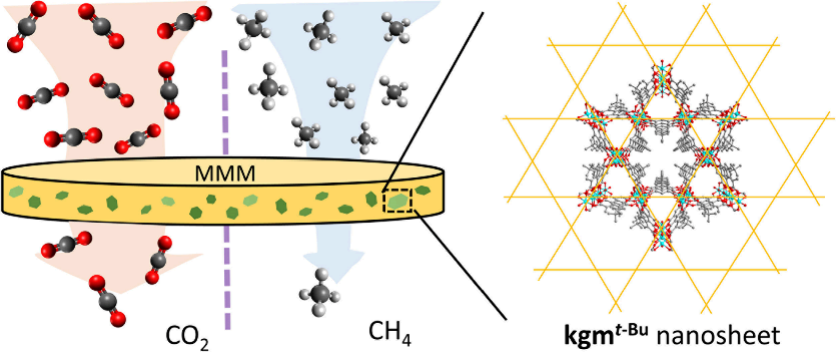

Mixed matrix membranes represent an important technology for gas separations. Nanosheets of metal–organic framework (MOF) materials of high aspect ratio and size-selective gas transport properties have the potential to promote the efficient mixing of components to form membranes for gas separation. Herein, we report a bottom-up synthesis of extended sheets of kagomé (kgm) topology, **kgm**^*t*-Bu^, via the linkage of [Cu_2_(O_2_CR)_4_] paddlewheels with 5-*tert*-butylisophthalic acid. The growth of the layered structure can be controlled by the choice of solvent and modulator. Nanosheets of **kgm**^*t*-Bu^ of average thickness of 20 nm and aspect ratio of 40 to 50 can be obtained, and the sieving effect of the channels in **kgm**^*t*-Bu^ boost the efficient separation of CO_2_ over CH_4_. A mixed matrix membrane comprising **kgm**^*t*-Bu^ nanosheets with Matrimid shows a 32% enhancement in CO_2_/CH_4_ selectivity compared with the membrane incorporating the MOF in the particulate form.

## 1 Introduction

Membrane-based gas separation is considered advantageous over energy-intensive separation techniques such as distillation.^1,2^ However, conventional polymeric gas separation membranes are limited by the intrinsic trade-off between the gas permeability and selectivity often described by the gas-pair dependent upper bounds.^3,4^ Porous materials such as zeolites and metal–organic framework (MOF) materials demonstrate enhanced gas separation performance well beyond the upper bounds of membranes.^5^ However, preparations of stand-alone membranes of these porous materials are nontrivial due to their crystalline nature.^6^ As an alternative, mixed matrix membranes (MMMs) afford a practical strategy in utilizing porous materials for high-performance gas separation membranes by distributing particles of porous material within a continuous polymer matrix.^7^

MOFs have been investigated as efficient solid physisorbents due to their selective adsorption and transport properties, and this has attracted interest in transferring these properties into MMMs for membrane-based gas separations.^8−10^ However, inadequate mixing between the polymer matrix and MOF can lead to the formation of heterogeneous phases and performance-deteriorating defects.^11^ More efficient mixing within membranes may be achieved by functionalization and shaping of MOF particles.^12,13^ Recently, MOFs with nanosheet morphology have received much attention in the preparation of MOF/polymer MMMs.^14−16^ These nanosheets are characterized by a high aspect ratio of lateral dimensions with respect to thickness, as well as aligned pores along a specific crystallographic direction due to the preferred orientation. Nanosheets of MOFs can be prepared top-down via exfoliation of its bulk material using sonication,^17^ ball milling,^18^ or intercalation.^19^ Alternatively, they can be synthesized bottom-up from their respective building blocks by exploiting synthetic methodologies in a controllable manner to form nanosheets of desired size and shape.^14,15,20−23^ Nanosheets derived from both 2D layered and nonlayered MOFs have been prepared by methods including diffusion-modulated growth,^14^ surfactant-assisted synthesis,^15^ and crystal growth control.^22^ Unlike the top-down approach that relies on preordered layered MOF structures, the bottom-up approach allows facile manipulation of the exposed facet and channel alignment along with controllable size and shape of the resultant material. For example, [001]-oriented nanosheets of the pillar-layered AlFFIVE-1-Ni can be prepared by controlling the concentration of the pillaring component and temperature.^22^ Nanosheets of (001)-AlFFIVE-1-Ni have been incorporated and aligned in-plane within polyimide matrices, where the 1D channels of the MOF structure lie in the [001] direction. As a result, the nanosheet effectively provides a short gas diffusion pathway with molecular sieving effects leading to MMMs with outstanding CO_2_/CH_4_ and H_2_S/CH_4_ separation performance at up to 60 wt % MOF loading.^22^

Herein, we report the synthesis of a layered MOF, named **kgm**^*t*-Bu^ (**kgm** = **kagomé**), constructed by reaction of Cu(II) salts with 5-*tert*-butylisophthalic acid (5-*t-*Bu-H_2_ip). The structure comprises undulating metal–ligand monolayers of **kgm** topology, and these stack in an AA fashion to form two distinct 1D channels with a narrowest opening of 3.2 Å. Inspired by the solvent-induced interlayer separation effect of dimethylformamide (DMF) on isostructural MOFs,^24^ nanosheets of **kgm**^*t*-Bu^ (*ns*-**kg m**^*t*-Bu^) were obtained by using 1:1 DMF/MeOH as a solvent to slow down the packing of layers. In addition, acetic acid was added to modulate the crystal growth via linker exchange, and this led to the formation of *ns*-**kgm**^*t*-Bu^ with a thickness of ca. 20 nm and aspect ratios of 40–50. Notable differences in morphology, surface area, and gas adsorption properties were observed between the bulk particles of **kgm**^*t*-Bu^ (*bp*-**kgm**^*t*-Bu^) and *ns*-**kg m**^*t*-Bu^. MMMs prepared with *ns*-**kgm**^*t*-Bu^ and Matrimid exhibit superior CO_2_/CH_4_ selectivity over both the bare polyimide membrane and the membranes prepared from *bp*-**kg m**^*t*-Bu^ and Matrimid. Molecular dynamics simulations of the diffusivities of CO_2_ and CH_4_ within **kgm**^*t*-Bu^ unveiled a size-screening effect originating from the distinct channels formed by the nanosheets of **kgm**^*t*-Bu^.

## 2 Experimental Methods

### 2.1 Chemical and Materials

Cu(NO_3_)_2_·3H_2_O, 5-*tert*-butylisophthalic acid (5-*t-*Bu-H_2_ip), benzimidazole, pyridine, and chloroform (CHCl_3_) were purchased from Sigma-Aldrich, Cu(CH_3_COO)_2_·H_2_O from Alfa Aesar, and *N*,*N*-dimethylformamide (DMF), acetic acid (AcOH), ethanol (EtOH), and methanol (MeOH) from Fisher Scientific. All chemicals were used as received without any further treatment. Polyamide (PI) Matrimid 5218 was purchased from Huntsman Advanced Materials and activated under dynamic vacuum at 453 K overnight to remove adsorbed moisture prior to the preparation of membranes.

### 2.2 Synthesis of Single Crystals of **kgm**^*t*-Bu^

Single crystals of **kgm**^*t*-Bu^ (*sc*-**kgm**^*t*-Bu^) were prepared via slow diffusion according to a modified procedure.^25^ A solution of DMF and EtOH (1:1, 3 mL) containing 5-*t-*Bu-H_2_ip (11 mg, 0.05 mmol) and benzimidazole (3.0 mg, 0.025 mmol) was placed at the bottom of an 8 mL Wheaton vial. An aqueous solution (1.5 mL) of Cu(NO_3_)_2_·3H_2_O (12 mg, 0.05 mmol) was layered onto the ligand. The vial was sealed and left upright at room temperature for over a week to give light blue hexagonal crystalline plates.

### 2.3 Synthesis of Bulk *bp*-**kgm**^*t*-Bu^

Bulk powder of **kgm**^*t*-Bu^ (*bp*-**kgm**^*t*-Bu^) was prepared by mixing and stirring a solution of DMF/MeOH (1:1, 20 mL) containing Cu(NO_3_)_2_·3H_2_O (240 mg, 1 mmol) and 5-*t-*Bu-H_2_ip (222 mg, 1 mmol) with pyridine (81.7 μL, 1 mmol) for 24 h at room temperature. A blue precipitate was obtained via centrifugation and washed 3 times with DMF to remove unreacted ligands and metal salts. Residual DMF and pyridine were removed by solvent exchange multiple times with acetone. Light blue *bp*-**kgm**^*t*-Bu^ (28 mg) was obtained as a powder after drying in the air. Element analysis was performed for [Cu(5-*t-*Bu-ip)(H_2_O)]·0.5CH_3_OH or C_12.5_H_16_O_5.5_Cu. Calcd (%), C: 47.23, H: 5.03, Cu: 20.01. Found (%), C: 47.35, H: 4.92, Cu: 19.08.

### 2.4 Synthesis of Nanosheet *ns*-**kgm**^*t*-Bu^

Cu(CH_3_COO)_2_·H_2_O (50 mg, 0.25 mmol) and 5-*t-*Bu-H_2_ip (55 mg, 0.25 mmol) were each dissolved in DMF/MeOH (1:1, 20 mL). Acetic acid (143 μL; 10 mol equiv) was added as modulator to the solution of 5-*t-*Bu-H_2_ip, and the solution of metal salt was added dropwise into it over 30 min from a dropping funnel under stirring. The solution became cloudy after about 1 h and was left stirring for 24h. The precipitate was collected and washed following the same procedure as for the bulk sample above. The blue powder product (11.3 mg) was obtained after drying in air and then dispersed and stored under CHCl_3_ for further use. For the preparation of membranes, the nanosheets were activated by heating at 453 K to remove residual solvent and moisture.

## 3 Results and Discussion

### 3.1 Synthesis and Structure of Single Crystals and Bulk Powder of **kg m**^*t*-Bu^

**Figure 1:**
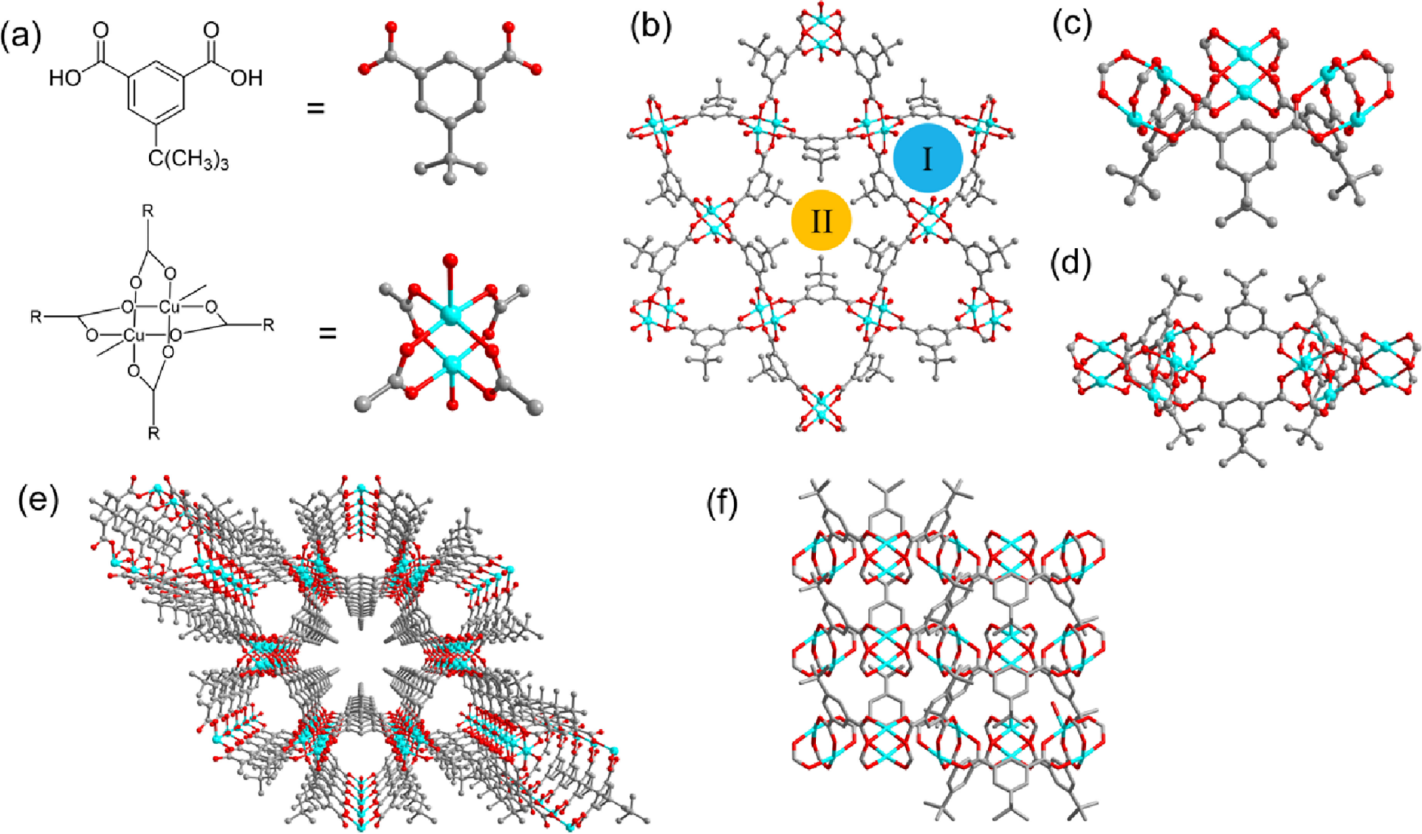
Views the structure of **kgm**^*t*-Bu^. Views of (a) the structure of the linker and [Cu_2_(OOCR)_4_] paddlewheel, (b) the monolayer showing the two types of channels, (c) the triangular motif, and (d) the hexagonal motif consisting of [Cu_2_(OOCR)_4_] paddlewheel and linkers. View of (e) the projection of the layered structure along the *c* axis and (f) the AA stacking of layers along *c*-axis (viewed in the *ab* plane). Color: carbon, gray; oxygen, red; copper, cyan. Hydrogen and oxygen atoms of axial coordinated water on [Cu_2_(OOCR)_4_] paddlewheels have been omitted for clarity.

Single crystals of **kgm**^*t*-Bu^ (*sc*-**kgm**^*t*-Bu^) were prepared by layering an aqueous solution of Cu(NO_3_)_2_ over a layer of DMF/EtOH (v/v = 1/1) containing the linker 5-*t-*Bu-H_2_ip (Figure 1a) and benzimidazole as a modulator. Slow diffusion and mixing of Cu(II) ions and the linker results in light blue hexagonal single crystals after 3 days. The single-crystal X-ray structure of [Cu_2_(C_12_H_15_O_4_)_2_(H_2_O)_2_] confirms the formation of a layered structure of **kgm** topology, isostructural with other reported **kgm** materials (Figure 1b).^24,26^ Within the monolayer, three [Cu_2_(OOCR)_4_] paddlewheels are linked by three linkers to form a triangular frustum-shaped motif. A secondary pseudohexagonal motif is formed by six surrounding triangular motifs to create a pocket space with a 4.8 Å maximum diameter (Figure 1c). The triangular motifs in the monolayer point alternatively in opposite directions, and this results in an undulated structure (Figure 1e) with neighboring layers stacked upon each other to form two types of channels. Channel I is formed via the stacking of the triangular motifs, featuring a polar surface decorated with O-centers from carboxylates and the coordinated water molecule on the [Cu_2_(OOCR)_4_] paddlewheel. Channel II is formed via stacking of the pseudohexagonal motifs, possessing a nonpolar internal surface due to surrounding *tert*-butyl groups from the linker. Both channels adopt irregular interior shapes as an array of stacking cones. The aperture of both channels is constrained by the hydrogen atoms of the linker, with the narrowest opening in both channels measuring approximately 3.2 Å in diameter based upon the crystallographic structure (Figure S6).

**Figure 2:**
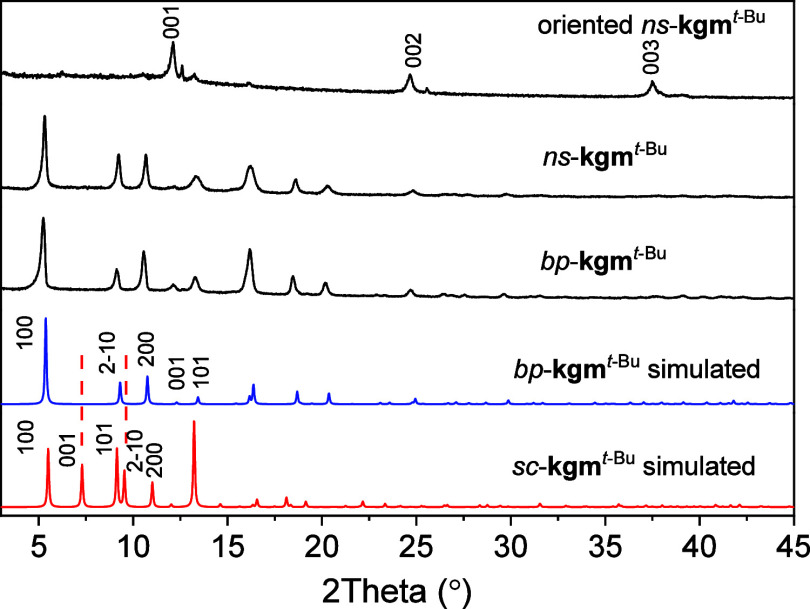
PXRD patterns of *ns*-**kgm**^*t*-Bu and^*bp*-**kgm**^*t*-Bu^ and the oriented pattern of nanosheets with the simulated pattern based upon the structural model obtained from refinement of the structure of *sc*-**kgm**^*t*-Bu^.

**Figure 3:**
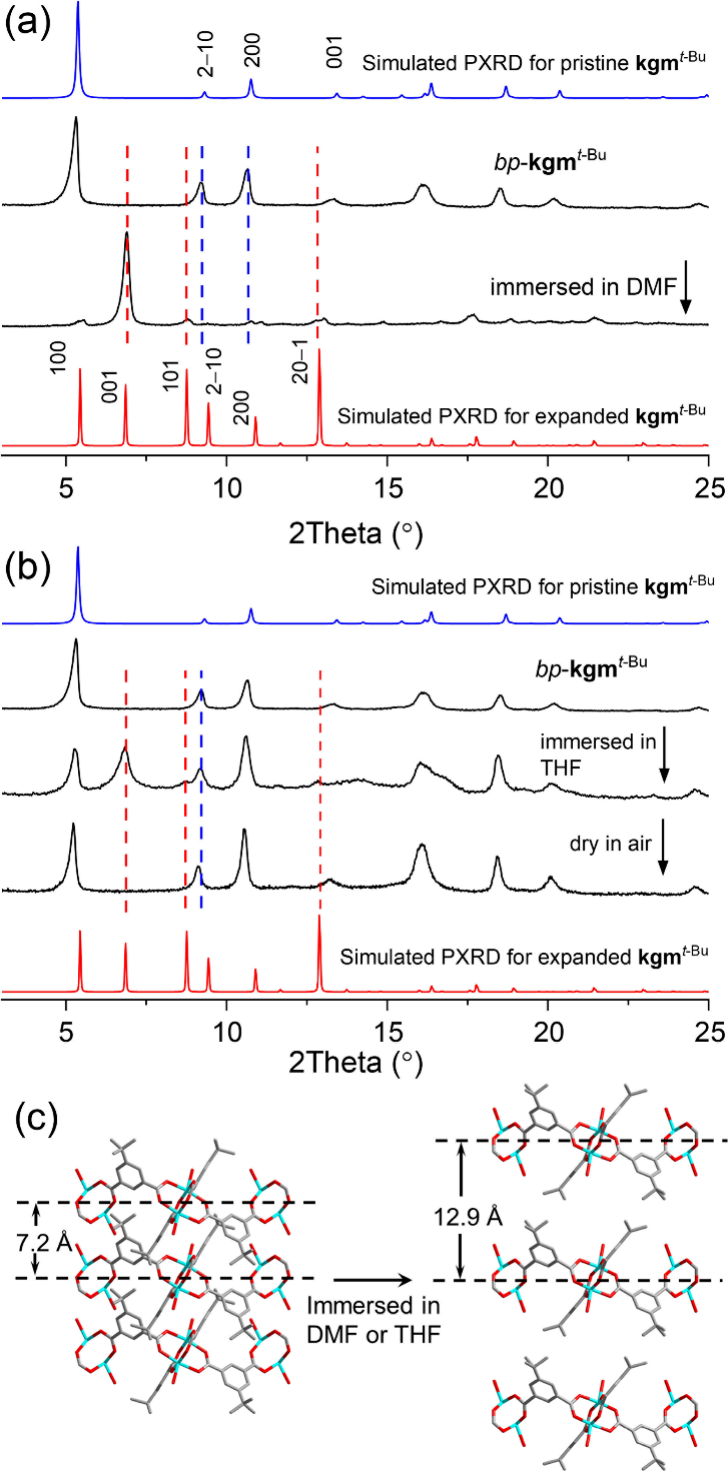
Interlayer expansion of the structure of **kgm**^*t*-Bu^. Effects of (a) DMF and (b) THF on the PXRD patterns of *bp*-**kgm**^*t*-Bu^ and (c) schematic illustration of the solvent-induced interlayer expansion.

Bulk *bp*-**kgm**^*t*-Bu^ was obtained by mixing equimolar quantities of Cu(NO_3_)_2_·3H_2_O and 5-*t-*Bu-H_2_ip in the presence of 1 equiv of pyridine in DMF/MeOH (v/v = 1/1). Powder X-ray diffraction (PXRD) patterns of *bp*-**kgm**^*t*-Bu^ show that peaks at 7.3° and 9.5° are missing compared to the simulated pattern from the single crystal structure (Figure 2). Le Bail refinement^27^ of the PXRD pattern using the trigonal space group *P*-3m1(No. 164) yielded a set of unit cell parameters [*a* = 18.7298(12), *c* = 7.0647(6) Å], which differ from those of the single crystal structure [*a* = 18.5264(3), *c* = 12.1041(2) Å]. The shorter *c* axis suggests that the metal–ligand layers pack more tightly in the powder structure than in the single crystal structure, and this was confirmed by the crystal structure of *bp*-**kgm**^*t*-Bu^ obtained by the Rietveld refinement of synchrotron PXRD data. Unlike *sc*-**kgm**^*t*-Bu^ where the adjacent monolayers interact through weak van der Waals forces, the monolayers in *bp*-**kgm**^*t*-Bu^ interact strongly via hydrogen bonds formed between the axial water on the [Cu_2_(OOCR)_4_] paddlewheel units with an O···O distance of 3.17(6) Å). As inspired by previous studies on solvent-induced interlayer separation,^24^ the effect of solvent on the layered structure was investigated. Two new PXRD peaks at 6.9° and 8.8° appeared on immersing *bp*-**kgm**^*t*-Bu^ in DMF, indicating an expansion of unit cell parameters to *a* = 18.74(3), *c* = 12.89(2) Å], close to the lattice parameters derived from the single crystal X-ray structure (Figure 3a). Similar features were reproduced when immersing *bp*-**kgm**^*t*-Bu^ in THF. Interestingly, the interlayer expansion can be reversed on drying the THF-immersed sample in air (Figure 3b). This suggests that close packing of monolayers in the presence of polar solvent such as DMF and THF and that interlayer separation can thus be controlled by the choice of solvent mix.

### 3.2 Synthesis and Structure of *ns*-**kgm**^*t*-Bu^

A bottom-up synthesis method was adopted to form nanosheets of the layered MOF. Previously reported **kgm** MOFs have used MeOH and pyridine as the solvent and modulator, respectively, in their synthesis.^24,26^ However, direct mixing of these components leads to rapid precipitation of bulk *bp*-**kgm**^*t*-Bu^, so a methodology to control the crystal growth was required in order to generate nanosheets of *ns*-**kgm**^*t*-Bu^. [Cu(OAc)_2_] incorporating a preformed [Cu_2_(OOCR)_4_] paddlewheel structure was therefore used with acetic acid as a modulator to control carboxylate linker-exchange rate between the carboxylates in [Cu(OAc)_2_] and 5-*t-*Bu-H_2_ip.^12,28,29^ Based on the above observations on the solvent effects on the observed layered structures, a 1:1 mixture of DMF and MeOH was used as the solvent to control the packing of monolayers in favor of nanosheet formation.

The PXRD pattern for *ns*-**kgm**^*t*-Bu^ showed peak positions matching those of *bp*-**kgm**^*t*-Bu^ (Figure 2). An oriented PXRD sample of *ns*-**kgm**^*t*-Bu^ was prepared by placing drops of nanosheet suspension onto zero background plates to investigate the preferred orientation of the nanosheets. The out-of-plane pattern of the oriented *ns*-**kgm**^*t*-Bu^ sample showed 3 main peaks at 12.1°, 24.6°, and 37.4° corresponding to the (001), (002), and (003) planes, respectively, suggesting that the monolayers in *ns*-**kgm**^*t*-Bu^ stack preferentially along the *c-*axis aligned with the channel openings.

**Figure 4:**
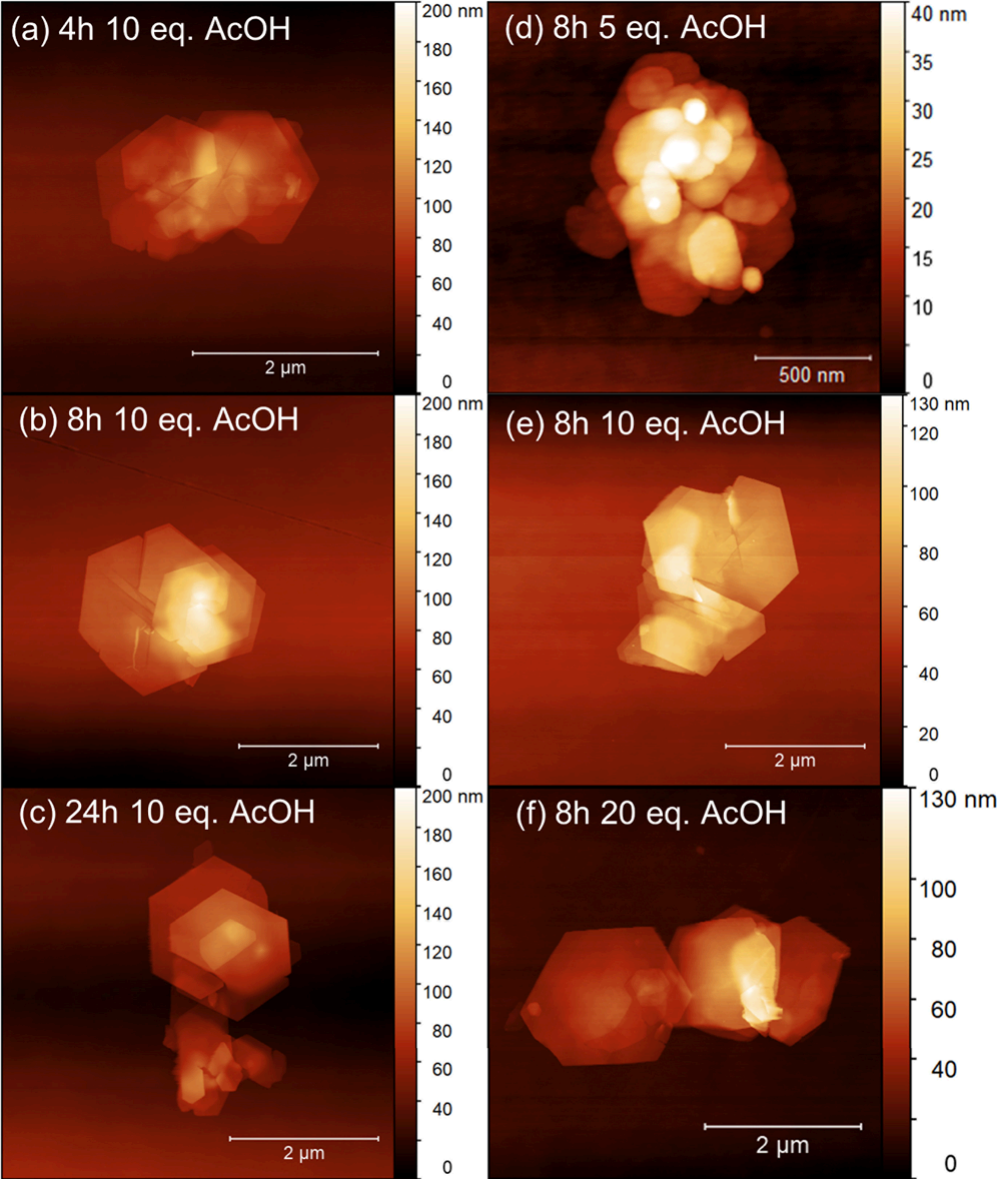
Effect of reaction time and acetic acid modulator on the thickness of *ns*-**kgm**^*t*-Bu^. AFM height images of nanosheets prepared with reaction times of (a) 4 h, (b) 8 h, and (c) 24 h in the presence of 10 equiv of acetic acid. Views of nanosheets prepared with (d) 5 equiv, (e) 10 equiv, and (f) 20 equiv of acetic acid at the same reaction time of 8 h.

The effects of reaction time and concentration of modulator on the preparation of *ns*-**kgm**^*t*-Bu^ were investigated and optimized by monitoring the dimensions of the product using atomic force microscopy (AFM). For *ns*-**kgm**^*t*-Bu^, by increasing the reaction time from 4 to 8 h at a fixed concentration of acetic acid at 10 equiv, the obtained nanosheets increase in thickness from an average of 15.7 to 19.3 nm (Figure 4a,b). However, prolonging the reaction time to 24 h resulted in a negligible change in the observed thickness (Figure S3a) nor lateral dimensions of the nanosheets (Figure S3c). The concentration of the acetic acid modulator shows notable impacts on both the thickness and lateral dimensions of the nanosheets (defined by the length of the hexagonal edge). Using 5 equiv of acetic acid in the synthesis afforded sheets of an average thickness of 8.5 nm and lateral dimensions of less than 500 nm. Increasing to 10 equiv of acetic acid led to an increase in thickness to 19.3 nm with lateral dimensions extended to 870 nm. At 20 equiv of acetic acid the dimensions were only slightly increased to 19.9 nm in thickness with lateral dimensions of 939 nm, within the error of nanosheets prepared with 10 equiv of modulator (Figure S3b). These results confirm that acetic acid functions as a structure-directing agent, promoting the growth of monolayers along the *ab*-plane. This modulator, in combination with the mixed solvents, effectively regulate the packing of monolayers and facilitate the formation of nanosheets with aspect ratios of 40–50. Using 10 equiv of acetic acid and a reaction time of 24 h resulted in a reproducible synthesis of *ns*-**kgm**^*t*-Bu^ with stable morphology and low thickness, and this form was thus chosen in the preparation of MMMs.

### 3.3 Characterization of *ns*-**kgm**^*t*-Bu^ and *bp*-**kgm**^*t*-Bu^

**Figure 5:**
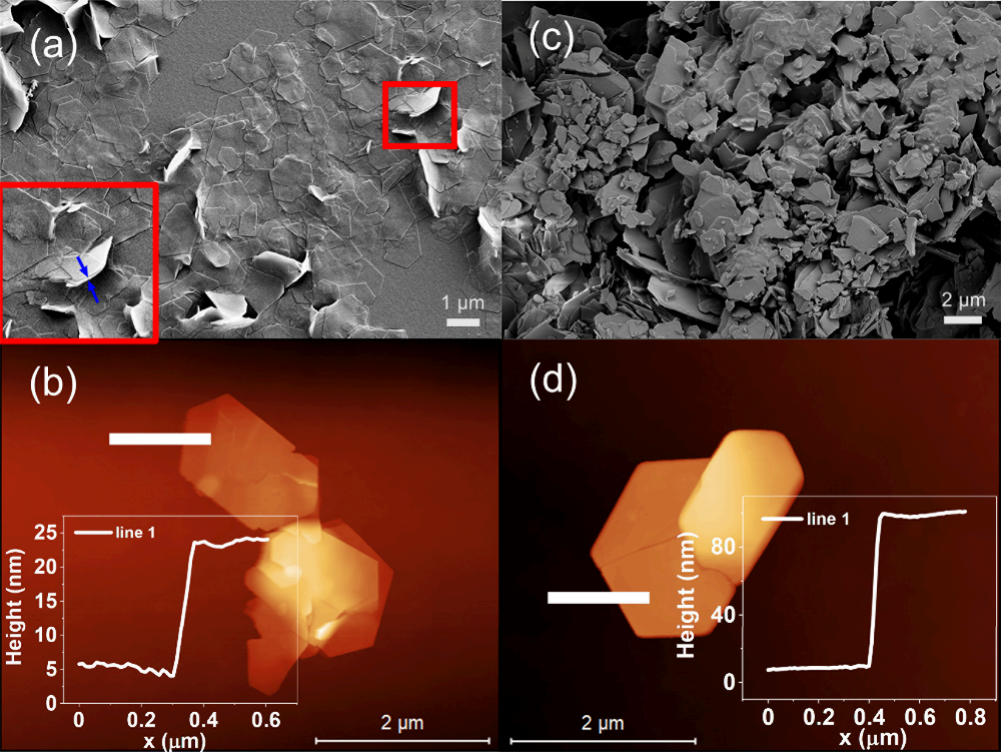
SEM images of (a) *ns*-**kgm**^*t*-Bu^ and (c) *bp*-**kgm**^*t*-Bu^. AFM height images of (b) *ns*-**kgm**^*t*-Bu^ and (d) *bp*-**kgm**^*t*-Bu^ with insets of height profiles along marked lines.

**Figure 6:**
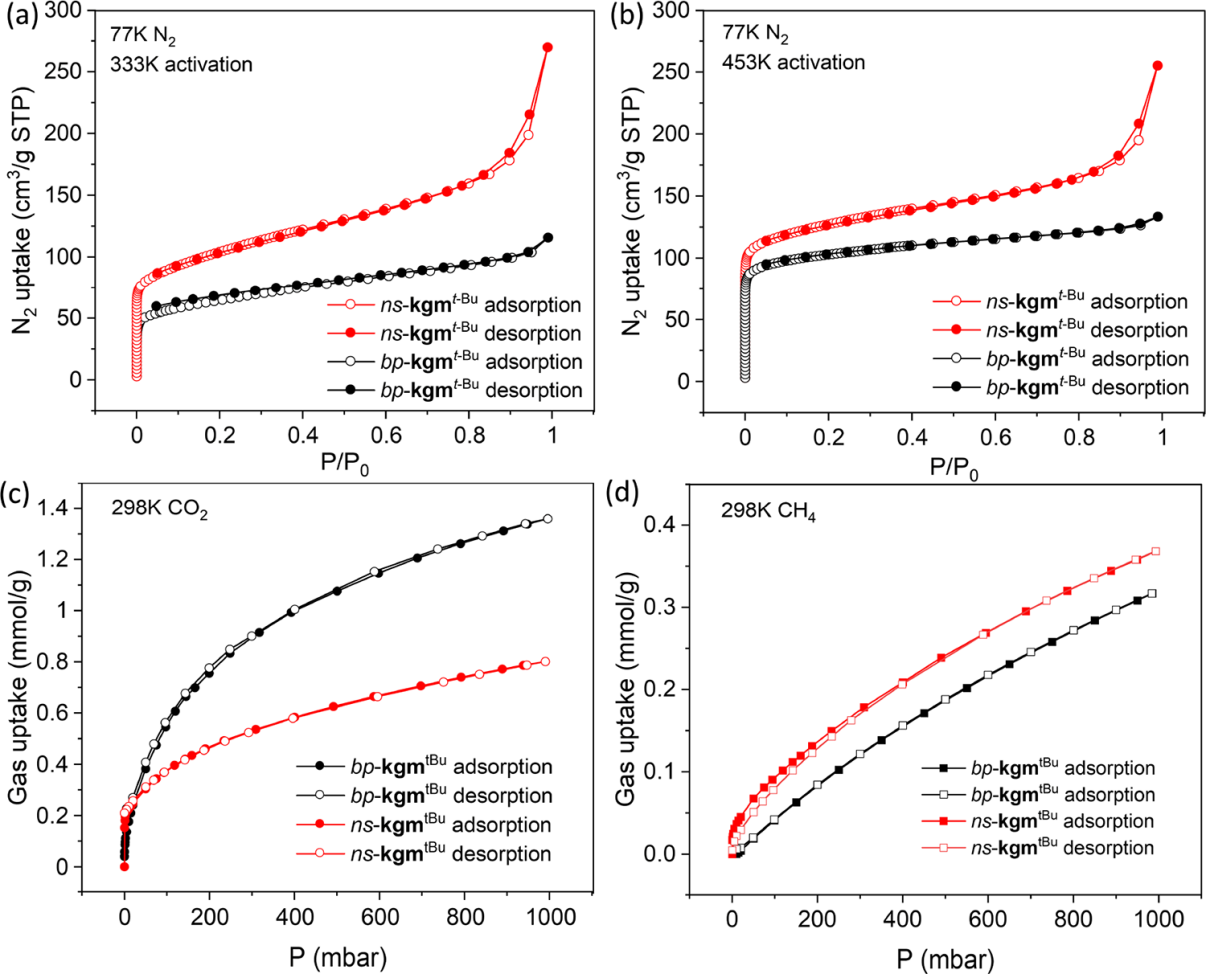
Adsorption isotherms measured at 77 K for N_2_ in *ns*-**kgm**^*t*-Bu^ and *bp*-**kg m**^*t*-Bu^ activated at (a) 333 K and (b) 453 K. Adsorption isotherms of (c) CO_2_ and (d) CH_4_ in *ns*-**kgm**^*t*-Bu^ and *bp*-**kgm**^*t*-Bu^ at 298 K.

The SEM/TEM images of *ns*-**kgm**^*t*-Bu^ and *bp*-**kgm**^*t*-Bu^ show clear differences in morphology: *bp*-**kgm**^*t*-Bu^ exhibits thick plates in random shapes with a thickness of over 100 nm as measured by AFM (Figure 5c,d). In comparison, *ns*-**kgm**^*t*-Bu^ shows hexagonal features (Figure 4) with a much lower thickness of an average 20 nm and high aspect ratio of 40–50 (Figure 5a,b, S4). The N_2_ adsorption isotherms for *ns*-**kgm**^*t*-Bu^ and *bp*-**kgm**^*t*-Bu^ at 77K were measured after activation at 333 K, and the Brunauer–Emmett–Teller (BET) surface areas (*S*_BET_) extracted from these isotherms were determined as 368 and 232 m^2^ g^–1^, respectively (Figure 6a). Activation of the materials at 453 K removed the coordinated water molecules on the [Cu_2_(OOCR)_4_] paddlewheel, and *S*_BET_ further increased to 468 and 386 m^2^ g^–1^ for *ns*-**kgm**^*t*-Bu^ and *bp*-**kgm**^*t*-Bu^, respectively (Figure 6b). The *T*-plot analysis of the N_2_ isotherms at 77 K afford the external surface area and pore volume for the samples activated at 453 K. The material *ns*-**kgm**^*t*-Bu^ displays an external surface area of 179 m^2^ g^–1^ compared with *bp*-**kgm**^*t*-Bu^ at 91.3 m^2^ g^–1^, while the micropore volume are similar between the two samples (0.1189 vs 0.1196 cm^3^ g^–1^, respectively). Thus, the high aspect ratio of *ns*-**kgm**^*t*-Bu^ contributes significantly to the higher N_2_ adsorption. An H1-type hysteresis was observed for *ns*-**kgm**^*t*-Bu^ at high relative pressure (*P*/*P*_0_ > 0.7) and assigned to interparticle porosity formed by the stacking of the nanosheets. Pore size distributions measured using the Horvath–Kawazoe method were obtained for the 333 K activated sample (Figure S5). For both samples, the peaks centered around 4 Å reflect the smaller pore of channel I with the larger pore contributing from the pseudohexagonal motif.

The different morphologies observed in this work impact the gas adsorption performance for CO_2_ and CH_4_ uptake and selectivity. Gravimetric adsorption isotherms confirm that *bp*-**kgm**^*t*-Bu^ shows uptakes for CO_2_ and CH_4_ of 1.36 and 0.32 mmol g^–1^, respectively, at 1 bar and 298 K with uptakes for *ns*-**kgm**^*t*-Bu^ of 0.80 and 0.37 mmol g^–1^, respectively, Figure 6c,d. Ideal adsorption solution theory (IAST) analysis for an equimolar mixture CO_2_/CH_4_ at 1 bar shows a selectivity of 18.5 (using the dual-site Langmuir Freundlich model) for *ns*-**kgm**^*t*-Bu^, which is much lower than the 108.4 (derived from dual-site Langmuir Freundlich model) and 50.1 (derived the single-site Langmuir Freundlich model) for *bp*-**kgm**^*t*-Bu^ (Figure S7). Given the identical chemical compositions, these differences closely relate to the morphologies of the isolated materials. The layered structure of **kgm**^*t*-Bu^ is constructed of monolayers stacked along the *c* axis in an AA mode. Thus, the thickness of the particle is directly related to the number of stacking monolayers, which determines the channel length and ultimately the available pore volume for gas adsorption. For CO_2_, the bulk powder has slightly higher pore volume and smaller external surface area, leading to a higher CO_2_ uptake. For CH_4_, both channels of the layered structure have limiting pore diameters lower than the kinetic diameters of CH_4_, suggesting that the adsorption of CH_4_ is primarily on the external surface. As *ns*-**kgm**^*t*-Bu^ possesses higher external surface area, the CH_4_ uptake is slightly increased compared to that of *bp*-**kgm**^*t*-Bu^, resulting in a lower CO_2_/CH_4_ adsorption selectivity.

Thermogravimetric analysis (TGA) was carried out for both *ns*-**kgm**^*t*-Bu^ and *bp*-**kgm**^*t*-Bu^. Upon increasing temperature in air, both samples lose adsorbed solvent/moisture (ca. 10 wt %) below 200 °C (Figure S8) with decomposition at around 300 °C to give a black powder at 600 °C. The molar ratio of linker to metal (L/M) calculated from the TGA curve (ca. 1.08) is consistent with a theoretical ideal value of 1.0 based upon the crystal structure.

### 3.4 MMMs for CO_2_/CH_4_ Separation

CO_2_ is commonly found in natural gas as an impurity that is not combustible and leads to acid formation. Thus, separation and removal of CO_2_ from CH_4_, the main component of natural gas, enhance the energy density of the gas and prevent pipeline corrosion. Improving the efficiency of separation membranes can also significantly reduce the cost of purification of natural gas. The potential of the layered **kgm**^*t*-Bu^ in membrane-based CO_2_/CH_4_ separation was evaluated by preparing MMMs using fillers of nanosheet and bulk powder morphologies. Nanoparticles of *ns*- or *bp*-**kgm**^*t*-Bu^ were dispersed in CHCl_3_ and mixed with a commercial polyimide matrix (Matrimid 5218) at mass loadings of 2–10 wt %. Membranes were cast via solvent evaporation.

**Figure 7:**
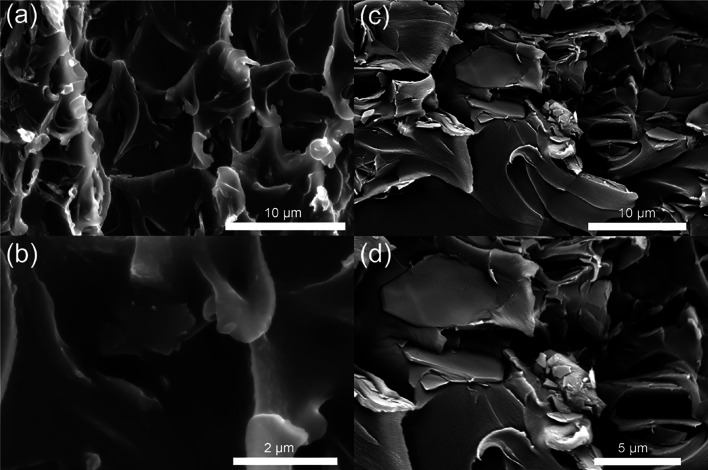
Cross-sectional SEM images of (a) and (b) *ns*-**kgm**^*t*-Bu^ Matrimid membranes, and of (c) and (d) 8 wt % *bp*-**kgm**^*t*-Bu^ Matrimid membranes.

**Figure 8:**
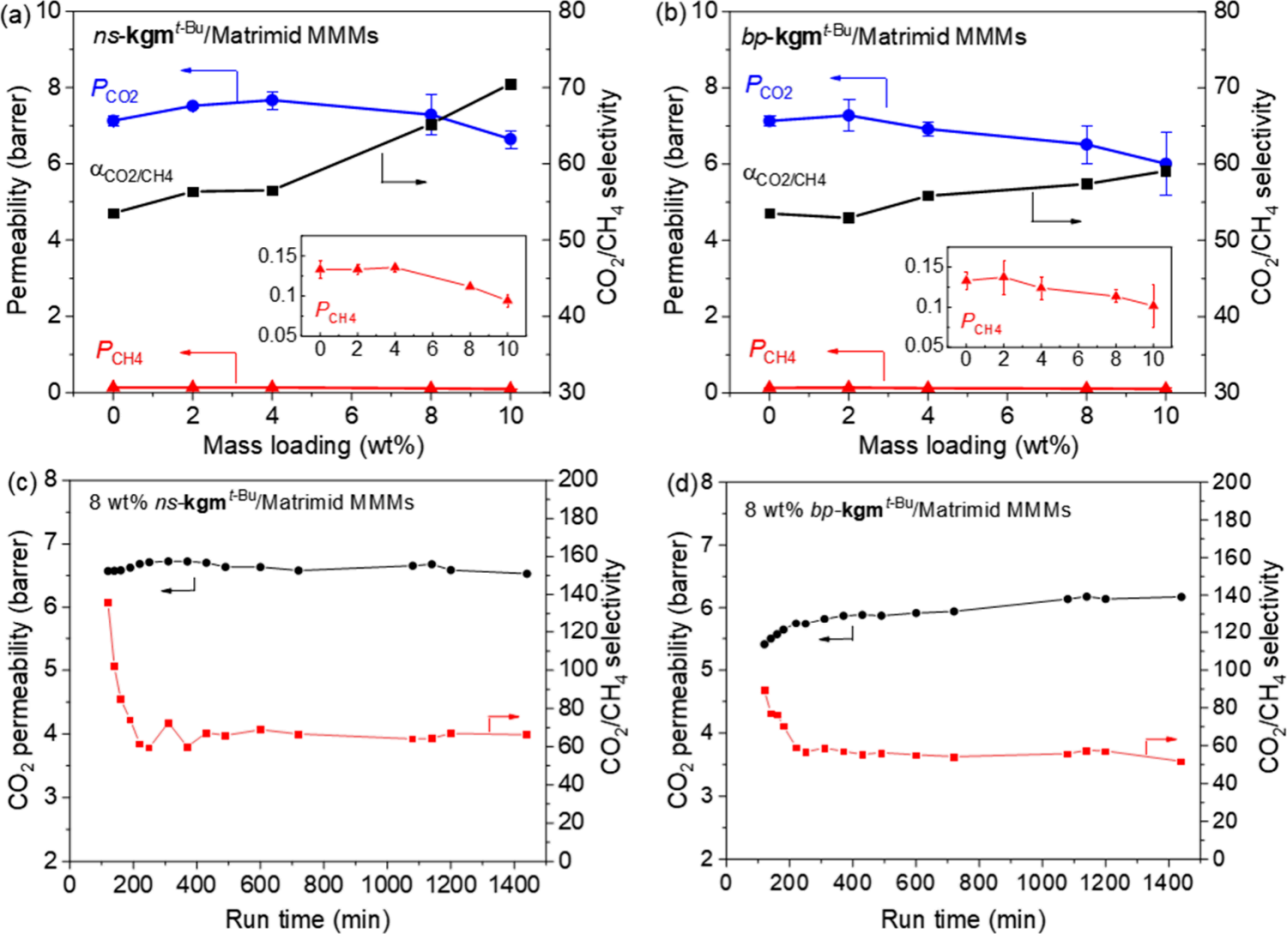
CO_2_/CH_4_ separation as a function of mass loading ratio of (a) *ns*-**kgm**^*t*-Bu^ Matrimid and (b) *ns*-**kgm**^*t*-Bu^ Matrimid MMMs. Long-term performance of (c) 8 wt % *ns*-**kgm**^*t*-Bu^ Matrimid and (d) 8 wt % *bp*-**kgm**^*t*-Bu^ Matrimid membranes.

The filler–polymer mixing efficiency in the MMMs was examined by using cross-sectional SEM images. Agglomerated particles were observed in the cross section of the membrane prepared from 8 wt % *bp*-**kgm**^*t*-Bu^ Matrimid but were absent for the *ns*-**kgm**^*t*-Bu^/polymer membrane (Figure 7). Gas permeabilities were obtained from a single gas permeation test through the membranes at a working pressure of 3 bar and effective surface area for the membrane of 19.6 cm^2^. The ideal separation selectivity was calculated from the ratio of individual gas permeabilities (*P*_CO2_/*P*_CH4_), and the effect of mass loading on separation was studied. At low mass loadings, the CO_2_ permeability steadily increased from 7.29 Barrer for the bare Matrimid membrane to 7.80 Barrer for the 4 wt % *ns*-**kgm**^*t*-Bu^ loaded Matrimid membrane. At higher loadings of *ns*-**kgm**^*t*-Bu^, the CO_2_ permeability decreased to 7.53 Barrer at 8 wt % loading and 7.05 Barrer at 10 wt % loading (Figure 8a). In comparison, the MMMs with *bp*-**kgm**^*t*-Bu^ displayed a monotonic decrease with lower CO_2_ permeabilities of 6.52 and 6.01 Barrer at 8 and 10 wt % loading, respectively (Figure 8b). The MMMs with the *ns*-**kgm**^*t*-Bu^ filler thus displayed overall higher CO_2_ permeabilities than MMMs with *bp*-**kgm**^*t*-Bu^. On the other hand, the CH_4_ permeability of *ns*-**kgm**^*t*-Bu^ Matrimid membrane decreased from 0.132 to 0.097 Barrer when increasing the loading from 2 wt % to 10 wt % (Figure 8a). Membranes with *bp*-**kgm**^*t*-Bu^ filler showed slightly higher CH_4_ permeability of 0.137 and 0.102 Barrer at 2 and 10 wt % loading, respectively (Figure 8b). In contrast, the bare Matrimid membrane exhibited a CH_4_ permeability of 0.140 Barrer. Consequently, the *ns*-**kgm**^*t*-Bu^ Matrimid membrane showed a CO_2_/CH_4_ selectivity of 70.5 at 10 wt % loading, which is notably higher than both the 10 wt % *bp*-**kgm**^*t*-Bu^ Matrimid membrane (59.1) and the bare Matrimid membrane (52.2). The separation performance of the 10 wt % *ns*-**kgm**^*t*-Bu^ Matrimid membrane (α_CO2/CH4_ = 70.5, *P*_CO2_ = 6.7) is among the best-performing MOF nanosheet Matrimid MMMs and is comparable to membranes prepared with nanosheets of CuBDC^16^^14^ and NH_2_-MIL-53(Al)^15^ with Matrimid (Table 1, Tables S5 and S6).

**Table 1:** Comparison of Performance of CO_2_/CH_4_ Separation of Reported MOF Nanosheets Matrimid MMMs

MOFs	loading/wt %	*P*_CO2_/Barrer	α_CO2/CH4_	ref.
CuBDC	8.2	4.1	78.7	(14)
CuBDC	12	6.3	38.0	(16)
NH_2_-MIL-53(Al)	8	13.3	31.1	(15)
NH_2_-MIL-53(Al)	16	13.5	34.4	
*ns*-**kgm**^*t***-Bu**^	0	7.1	53.5	this work
	8	7.3	65.2	
	10	6.7	70.5	
*bp*-**kgm**^*t***-Bu**^	8	6.5	57.4	
	10	6.0	59.1	

The superior CO_2_/CH_4_ separation selectivity of *ns*-**kgm**^*t*-Bu^ Matrimid MMMs was attributed to both the morphology of the nanosheet and the layered structure of the MOF. The nanosheet morphology shortens the pathway for molecular diffusion through the membrane, while the width of the smallest channel (3.2 Å) enables sieving of gas molecules. The kinetic diameter of CO_2_ (3.3 Å^30^) is very close to that of this narrowest channel, and the linear molecular geometry thus enables its diffusion through the channels, while transport of nonlinear CH_4_ molecules (3.8 Å^30^) is more limited. To compare the solution-diffusion process for different gases, Henry coefficients (*K*_H_) were calculated using a grand canonical Monte Carlo (GCMC) simulation to evaluate the affinity of the gases in **kgm**^*t*-Bu^. The layered MOF shows significantly higher affinity for CO_2_ (*K*_H,CO2_ = 5.3 × 10^–4^ mol kg^–1^ Pa^–1^) than CH_4_ (*K*_H,CH4_ = 3.2 × 10^–5^ mol kg^–1^ Pa^–1^), and molecular dynamic simulations on the self-diffusivity of CO_2_ and CH_4_ in **kgm**^*t*-Bu^ were also conducted (Figure S9). The mean squared displacement (MSD) of CH_4_ molecules in the framework reached a plateau only after a few picoseconds (ps), leading to a negligible CH_4_ diffusion coefficient (*D*_CH4_). In contrast, CO_2_ molecules diffuses freely with diffusion coefficients correlated with the number of CO_2_ molecule per unit cell. The value of *D*_CO2_ increased from 3.58 × 10^–12^ m^2^ s^–1^ (1 CO_2_ per cell) to 4.9 × 10^–11^ m^2^ s^–1^ (3 CO_2_ per cell) (Table S4), thus confirming the preferred sorption and diffusion of CO_2_ over CH_4_ within the layered MOF. It should be noted that *D*_CO2_ for Matrimid is between 10^–13^ – 10^–12^ m^2^ s^–1^.^31^ Thus, *D*_CO2_ in **kgm**^*t*-Bu^ was only slightly higher than that of the polymer matrix, which explains the limited improvement in permeability observed for these in MMMs.

The gas permeation performance after 24 h was tested for 8 wt % *ns*-**kgm**^*t*-Bu^ or *bp*-**kgm**^*t*-Bu^ loaded MMMs (Figure 8c,d). Both membranes initially displayed a transition stage during which the permeability of CO_2_ increased gradually and the CO_2_/CH_4_ selectivity dropped. This reflects the time required for equilibration due to the dense nature of the polymer membrane. This transition stage was more obvious in the MMMs with *bp*-**kgm**^*t*-Bu^ due to the lower CO_2_ permeability. The 8 wt % *ns*-**kgm**^*t*-Bu^ Matrimid membrane reached its highest CO_2_ permeability (ca. 6.7 Barrer) after around 5 h and this was maintained throughout the rest of measurements (Figure 8c). The membrane with *bp*-**kgm**^*t*-Bu^ at the same loading reached a slightly lower CO_2_ permeability of ca. 6.2 Barrer after 24 h. However, the *ns*-**kgm**^*t*-Bu^ MMM maintained a higher CO_2_/CH_4_ selectivity of 66.2, whereas the 8 wt % *bp*-**kgm**^*t*-Bu^ Matrimid membrane showed a lower selectivity of 51.8 after the extended run (Figure 8d). Thus, the *ns*-**kgm**^*t*-Bu^ Matrimid membrane showed a more consistent CO_2_/CH_4_ separation performance in comparison with that of the *bp*-**kgm**^*t*-Bu^ Matrimid membrane.

## 4 Conclusions

We have described the bottom-up synthesis of nanosheets of the layered MOF **kgm**^*t*-Bu^. The use of a polar solvents (DMF or THF) along with a modulator successfully controls crystal growth in favor of forming nanosheets with high aspect ratios. The downsizing of the layered structure into nanosheets significantly alters the adsorption performance and surface areas in comparison with its bulk counterpart. The channels in **kgm**^*t*-Bu^ function as size-selective molecular sieves with CO_2_ flowing more readily than CH_4_, as supported by simulation analysis. This affords enhanced CO_2_ permeability and higher CO_2_/CH_4_ selectivity in the *ns*-**kgm**^*t*-Bu^ Matrimid membranes compared with both the pure Matrimid membrane and *bp*-**kgm**^*t*-Bu^ Matrimid MMMs. Furthermore, the *ns*-**kgm**^*t*-Bu^ Matrimid membranes maintained their high selectivity and permeability in extended gas permeation tests.

## Supporting Information Available

Characterization methods including diffraction, AFM, TGA, TEM; membrane preparation and gas permeation test methods; structure and refinement and analysis of nanosheets; adsorption isotherms and IAST fittings; DFT and GCMC calculation methods and molecular dynamic simulations; single crystal structure of **kgm**^*t*-Bu^ (CCDC 2204427) and crystal structure of bulk powder of **kgm**^*t*-Bu^ (CCDC 2204003) are available free of charge from the Cambridge Crystallographic Data Centre (https://www.ccdc.cam.ac.uk/) (PDF)
