# Supplementary material for: Nanosheets of a Layered Metal–Organic Framework for Separation of CO2/CH4 using Mixed Matrix Membranes
Source: ACS Appl Mater Interfaces. 2024 Jun 13;16(25):32524–32. doi: 10.1021/acsami.4c05611 (PMC11212018; doi:10.1021/acsami.4c05611)
Supplement: Supplementary file 1 — am4c05611_si_001.pdf [file am4c05611_si_001.pdf]

# Supporting Information

# Nanosheets of a Layered Metal-Organic Framework for Separation of $CO_2$/$CH_4$ using Mixed Matrix Membranes

*Meng He[1], Yinlin Chen[1], Wanpeng Lu[1], Lixia Guo[1,2], Kui Hu[1], Xue Han[1,3], Inigo Vitorica-Yrezabal[1], Catherine Dejoie[4], Andrew N. Fitch[4], Martin Schröder[1]* and Sihai Yang[1,2]**

[1] Department of Chemistry, University of Manchester, Manchester, M13 9PL, UK

Email: M.Schroder@manchester.ac.uk; Sihai.Yang@manchester.ac.uk

[2] College of Chemistry and Molecular Engineering, Beijing National Laboratory for Molecular Sciences, Peking University, Beijing, 100871, China

Email: Sihai.Yang@pku.edu.cn

[3] College of Chemistry, Beijing Normal University, Beijing 100875, China

[4] The European Synchrotron Radiation Facility, 71 Avenue des Martyrs, CS40220, 38043 Grenoble Cedex 9, France

**List of contents**

**1. Materials and Methods** ....... 2

**1.1. Characterisation** ....... 2

**1.2. Membrane performance testing** ....... 3

**1.3. Calculations and simulations** ....... 4

**2. Single crystal structure of kgm$^{t\text{-}Bu}$** ....... 6

**3. Refinement of powder diffraction patterns** ....... 8

**4. Data for morphology of *ns*-kgm$^{t\text{-}Bu}$** ....... 10

**5. TEM image of *ns*-kgm$^{t\text{-}Bu}$** ....... 11

**6. Pore size distribution analysis** ....... 12

**7. Gravimetric adsorption isotherms and IAST analysis** ....... 13

**8. Thermogravimetric analysis** ....... 14

**9. Molecular dynamic simulations** ....... 15

**10. Comparison of gas separation performance of MMMs** ....... 16

**11. PXRD of *ns*-kgm$^{t\text{-}Bu}$ Matrimid MMMs** ....... 17

**12. Images of *ns*-kgm$^{t\text{-}Bu}$ Matrimid MMMs** ....... 18

## 1. Materials and Methods

### 1.1. Characterisation

**Single-crystal X-ray diffraction**. Single-crystal diffraction data for the complex[1] were collected at 100 K on a Rigaku FR-X diffractometer using Cu Kα radiation (λ = 1.5418 Å) equipped with a hybrid pixel array detector and an Oxford Cryosystems liquid $N_2$ flow system. Data collection, frame integration and data processing were performed using CrysAlisPro program suite. The structure was solved using SHELXT and refined on $F^2$ by full-matrix least-squares method using SHELXL within Olex2 suite.[2] All full occupancy non-hydrogen atoms were refined with anisotropic thermal displacement parameters. Positions of hydrogen atoms on methyl groups, aromatic rings, and coordinated water molecules on the $[Cu_2(OOCR)_4]$ paddlewheel were refined using riding coordinates.

**Powder X-ray diffraction.** Powder patterns over a 2θ range of 3-50° were obtained on a Panalytical X'Pert diffractometer using Cu Kα radiation (λ=1.54778 Å). The powder sample was mounted on a flat zero background plate and scanned in steps of 0.0167° in Bragg-Brentano geometry. For the oriented *ns*-**kgm**$^{t\text{-Bu}}$, the sample was prepared by allowing drops of a suspension of the nanosheet in $CHCl_3$ to dry in air on the plate.

**High-resolution powder diffraction and structure refinement.** High-resolution PXRD of the powder were collected at beamline ID22 of European Synchrotron Research Facility (ESRF) at Grenoble, France.[3] The sample was packed into a 0.7 mm capillary, which was sealed and mounted on a brass spinner. The sample was attached onto a goniometer head and aligned to the beam spot, and the diffraction pattern collected at a wavelength of 0.3542 Å. Pawley refinement was first carried out to extract unit cell parameters, and Rietveld structure refinement on atom positions was carried out using Bruke-AXS Topas (V5.0). The initial structure was simulated and modelled based on the DFT geometry optimization result(details below). The final structure solution was obtained with a good agreement ($R_{wp}$ = 5.966 %, $R_{exp}$= 3.182 % and GoF = 1.875).

**Atomic force microscopy (AFM)**. AFM images were collected on a Bruker Multimode8 AFM system with ScanAsyst-Air probe. Samples were prepared by placing a drop of the *bp*-**kgm**$^{t\text{-Bu}}$ or *ns*-**kgm**$^{t\text{-Bu}}$ dispersion on a clean Silicon wafer. The thickness of the nanosheet sample was obtained using the extracted line profile from the raw data using Gwyddion. The mean thickness and distribution plot were calculated based on the statistics of over 30 nanosheets.

**BET surface area.** The $N_2$ adsorption isotherms at 77 K were measured on a Micromeritics 3Flex adsorption analyser. The samples were first activated overnight under dynamic vacuum using Smart PrepVac device at the target temperature. A liquid nitrogen cooling bath was used for the isotherm measurement. The BET surface areas for *bp*-**kgm**$^{t\text{-Bu}}$ and *ns*-**kgm**$^{t\text{-Bu}}$ were fitted from the $N_2$ isotherm data. The points in the range of 0.01-0.1 ($P/P_0$) were selected for the linear fitting as commonly used for microporous materials.

**Gas adsorption isotherms.** Gravimetric adsorption isotherms of $CO_2$ and $CH_4$ at 298K were measured on an IGA gravimetric sorption analyser (Hidden Isochema, Warrington, UK). The samples were *in situ* activated overnight at 453 K under dynamic vacuum prior to adsorption measurement.

**Thermogravimetric analysis (TGA)**. TGA measurements were conducted on a TA SDT650 thermogravimetric analyser. The experiments were carried out under air at ambient pressure using ramping rate of 2 °C/min from room temperature to 600 °C. An empty aluminium plate was used as reference.

**Scanning Electron Microscopy (SEM).** SEM images of the MOF samples were obtained using a Quanta FEC 650 system. Powder samples were mounted on an adhesive carbon tape. For the nanosheet materials, the sample was prepared by placing drops of the suspension on a clean Si wafer, which was then pasted onto a stub using conductive carbon tape. For membranes, the cross-section sample was prepared using the freeze-fracture method with liquid $N_2$. All samples were further coated by Pt or Au to avoid surface charging.

**Transmission Electron Microscopy (TEM).** A JEOL JEM-2100F TEM system with operating electron beam at 200 kV was used for TEM imaging. The sample was prepared by placing drops of a dispersion of the nanosheet onto a lacey carbon film supported on a 200-mesh copper grid.

### 1.2. Membrane performance testing

**Prpearation of mixed matrix membranes (MMMs).** Membranes with and without MOF filler were prepared by a solvent evaporation method. A cast solution containing a total mass of 0.35 g (MOF powder and polymer) was prepared by dispersing *bp*-**kgm**$^{t\text{-Bu}}$ or *ns*-**kgm**$^{t\text{-Bu}}$ was dispersed in $CHCl_3$ (5 mL) in a glass vial. The suspension was sonicated for 30 mins to allow further exfoliation and achieve an even dispersion. Vacuum dried polymer powder was added slowly to the suspension under vigorous stirring. The cast solution was then sonicated for a further 15 mins and mixed on a roller mixer for another 15 mins at 60 rad/s. This sonication and mixing cycle were repeated twice to ensure good mixing. The membrane was prepared by pouring the viscous cast solution into a clean petri dish on a level surface inside an air-ventilated fume hood. The solvent was evaporated under a flow of air (0.4 $m^3$/s) for a day. The membrane was then peeled off and activated under dynamic vacuum at 383K overnight to remove residual solvent. The thickness of the activated membrane was measured using a digital micrometre and averaged over 15 locations.

**Gas permeation measurements.** Membranes were cut into a round shape and placed onto a porous stainless-steel support, which was then clapped between O-rings in a flange. Gases were introduced via mass flow controllers, and the pressure controlled *via* a metering valve. Permeate gas through the membrane was swept by a 20 sccm (standard cubic centimetres per minute) helium stream. A gas chromatography (Micro GC 490, Agilent Technology) equipped with 10m PoraPLOT U column was connected in-line through a three-way valve. The exhaust gas was extracted by the ventilation system. A minimum of 2 h was allowed for the

membrane to reach equilibrium before measuring its performance. The permeation test was conducted at an operating pressure of 3 bar and an effective area of 19.6 $cm^2$.

Performance of gas separation was evaluated by single gas permeabilities and selectivity. The permeability of a gas component ($P_i$) through the membrane is calculated according to equation (2):

$$P_i = \frac{N_i \cdot d}{A \cdot \Delta p} \qquad \text{eq. (2)}$$

where $N_i$ (mol/s) represent the molar flow rate of the component, $d$ (m) is the thickness of the membrane, $\Delta p$ (Pa) is the trans-membrane pressure difference and $A$ ($m^2$) is the membrane effective permeation area (19.6 $cm^2$). The molar flow rate was calculated based on the calibrated concentration curves of mixtures of gas component $i$ and carrier gas helium measured by GC.

Ideal selectivity of membrane towards different gas component was calculated based on the pure gas permeabilities according to equation (3):

$$\alpha = \frac{P_i}{P_j} \quad \text{eq. (3)}$$

where $\alpha$ is the selectivity, $P_i$ and $P_j$ are the permeability of different gas components $i$ and $j$.

### 1.3. Calculations and simulations

**DFT geometry optimisation of the initial structure.** Density functional theory (DFT) calculations were used in the optimisation of the modelled structure. The simulated structure was constructed by starting with the fractional coordinates obtained from the published structure of STAM-NMe2.[4] The lattice parameters were modified according to the Le Bail refinement results of the PXRD pattern. The pendant tert-butyl group was then connected by first changing the H atom bonded to the phenyl ring to carbon. The three $-CH_3$ groups were added by placing one of the C atoms on the mirror plane and the other two manually connected based on $sp^3$ hybridisation at the C centre. Hydrogen atoms on the methyl group were included. The atomic positions and unit cell parameters were optimised using the PWscf (plane-wave self-consistent field) package in Quantum ESPRESSO.[5,6] Periodic boundary conditions (PBC) were used along with a plane-wave basis set implemented in the software, and Perdew-Burke-Ernzerhof (PBE) exchange-correlation functionals were also incorporated. To speed up calculation, the core electrons for all elements were treated with Projector-Augmented Wave (PAW) pseudopotentials from the QE pseudopotential library. The charge density and wave function cutoff energies were set at 560 Ry and 70 Ry, respectively. Long range van der Waals interactions were corrected using the Grimme-D3 scheme (DFT-D3), and the Broyden–Fletcher–Goldfarb–Shanno (BFGS) algorithm was used for optimisation of both the lattice dimensions and atomic positions. The Brillouin zone was sampled by 1×1×2 Monkhorst-Pack k-point mesh for one unit cell. The convergence criteria for the SCF calculation and total force were set at $1\times10^{-5}$ and $1\times10^{-3}$ (a.u.), respectively.

**GCMC and molecular dynamics simulations.** All simulations were carried out using the RASPA2 software.[7,8] The structure from Rietveld refinement was used to create a desolvated **kgm**$^{t\text{-Bu}}$ framework by removing coordinated water from the $[Cu_2(OOCR)_4]$ paddlewheel. A supercell containing 16 unitcells (2×2×4)

was used with periodic conditions applied. The partial charge for the framework atoms were calculated using the built-in charge equilibration method. Grand canonical Monte Carlo (GCMC) simulations were performed to calculate the Henry's constant by running $1\times10^4$ cycles.

Molecular dynamic (MD) simulations were carried out to study the self-diffusivity of $CO_2$ and $CH_4$ within **kgm**$^{t\text{-Bu}}$ at 298K. The interaction between $CO_2/CH_4$ and the **kgm**$^{t\text{-Bu}}$ framework was described by van der Waals interaction (Lennard-Jones potential) and long-range Coulombic interactions. The cutoff radius for vdW interactions were set at 12 Å and the electrostatic interaction energy were summed using the Ewald method. The Universal forcefield (UFF) parameters were used for framework atoms (Cu, C, H, O), where $CO_2$ and $CH_4$ were described using the TraPPE forcefield. The partial charges for $CO_2$ were 0.7 e for C and −0.35 e for O, respectively. No electrostatic interaction was considered for $CH_4$. Simulations were carried out in the canonical ensemble (NVT) for 2 different loadings for $CH_4$ (1 and 2 molecules per unit cell) and 4 different loadings for $CO_2$ (1, 2, 3 and 4 molecules per unit cell). The Nose-Hoover thermostat was used by default to maintain the temperature, and a time step of 1 femtosecond (fs) was used in all calculations. The guest molecules were first randomly inserted into the host framework and underwent $1\times10^4$ cycles of Monte Carlo simulation to reach an equilibrium molecular arrangement. The initial velocity of all atoms was assigned according to the Maxwell-Boltzmann distribution at the target temperature. The system was then equilibrated for $1\times10^6$ NVT MD cycles prior to the $1\times10^6$ steps of production run, and the diffusion coefficients (*D*) calculated from the slopes of mean square displacement vs. time plots.

## 2. Single crystal structure of kgm$^{t\text{-Bu}}$

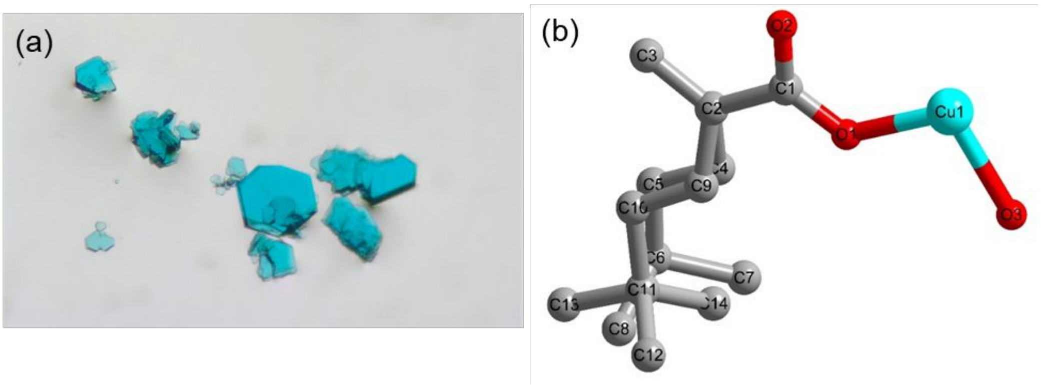


**Figure S1.** (a) View of crystals of *sc*-**kgm**$^{t\text{-Bu}}$ showing their hexagonal shape. The size of the largest crystal is around 50-60 µm. (b) Asymmetric unit of the single crystal structure of *sc*-**kgm**$^{t\text{-Bu}}$ at 100 K. The phenyl ring is disordered over two positions (C4/C9 and C5/C10) and the *tert*-butyl group disordered over three positions. The quaternary carbon is disordered between C6, C11 where the methyl groups are disordered between C7, C8, C12, C13 and C14.

**Table S1.** Crystallographic data for the single crystal structure of **kgm**$^{t\text{-Bu}}$ at 100K.

| Name | **kgm**$^{t\text{-Bu}}$ |
|---|---|
| CCDC number | 2204427 |
| Moiety formula | $C_{24}H_{28}Cu_2O_{10}$, $[Cu_2(L)_2(H_2O)_2]_n$ |
| Radiation | Cu Kα (λ=1.54184) |
| M, g·mol$^{-1}$ | 603.54 |
| Crystal system | Trigonal |
| Space group | P −3 m 1 (164) |
| a=b, Å | 18.5264(3) |
| c, Å | 12.1041(2) |
| α=β, deg | 90 |
| γ, deg | 120 |
| Volume, Å$^3$ | 3597.87(13) |
| $d_{calc}$, g/cm$^3$ | 0.836 |
| Z | 3 |
| F(000) | 930 |
| Crystal size, mm$^3$ | 0.06×0.06×0.02 |
| Density (calculated) | 0.836 |
| Absorption coefficient, mm$^{-1}$ | 1.347 |
| Theta range for data collection | 2.7110 to 65.9470° |
| Index ranges | -23<=h<=23, -20<=k<=18, -15<=l<=15 |
| Total reflections | 27956 |
| Goodness of fit | 1.035 |
| R indices (all) | R1=0.0682, wR2=0.2224 |

## 3. Refinement of powder diffraction patterns

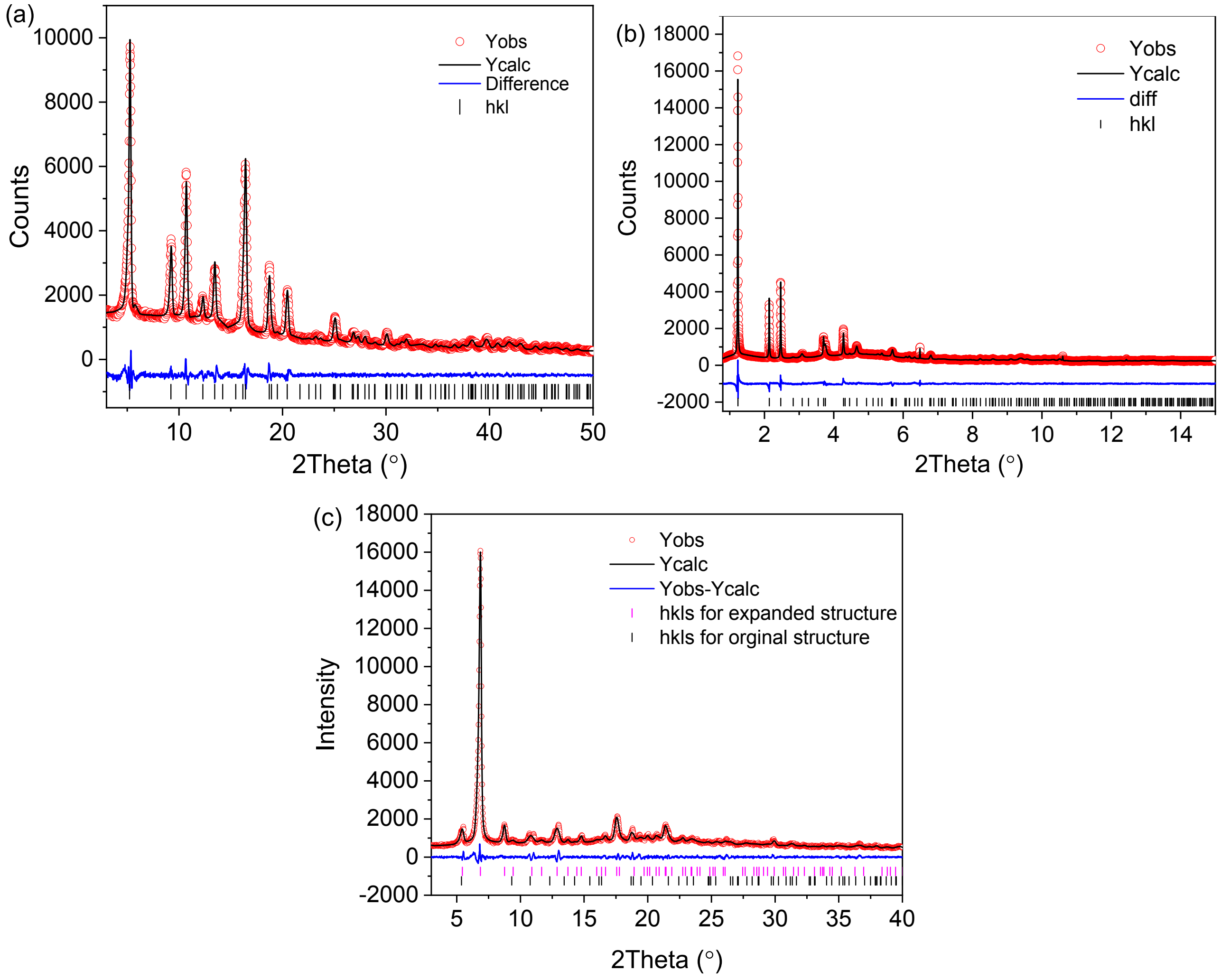


**Figure S2.** (a) Le Bail refinement of PXRD data for **kgm**$^{t\text{-Bu}}$. (b) Rietveld refinement of the synchrotron X-ray powder diffraction pattern and (c) Pawley refinement for DMF-immersed **kgm**$^{t\text{-Bu}}$.

**Table S2.** Le Bail refinement of PXRD data of the bulk **kgm**$^{t\text{-Bu}}$ powder

| Parameters | Value |
|---|---|
| Crystal system | Trigonal |
| Space group | P -3 m 1 (164) |
| a = b/Å | 18.7298(12) |
| c/Å | 7.0647(6) |
| α = β/ ° | 90 |
| γ/ ° | 120 |
| V/ ($10^6$ $pm^3$) | 2146.3(3) |
| Rwp | 5.12 |
| Rexp | 3.25 |
| Goodness of fit ($X^2$) | 2.48 |

**Table S3.** Rietveld refinement of high resolution synchrotron PXRD data for the bulk **kgm**$^{t\text{-Bu}}$ powder

| Parameters | Value |
|---|---|
| Name | *bp*-**kgm**$^{t\text{-Bu}}$ |
| CCDC number | 2204003 |
| Crystal system | Trigonal |
| Space group | P -3 m 1 (164) |
| Z | 6 |
| Moiety formula | C12 H12 Cu O6.63 |
| a = b / Å | 18.9723(3) |
| c / Å | 7.1865(17) |
| α = β / ° | 90 |
| γ / ° | 120 |
| V/ ($10^6$ $pm^3$) | 2240.2(5) |
| Number of refinable parameters | 61 |
| Rwp | 6.808 |
| Rexp | 3.182 |
| Goodness of fit ($X^2$) | 2.140 |

## 4. Data for morphology of *ns*-kgm$^{t\text{-Bu}}$

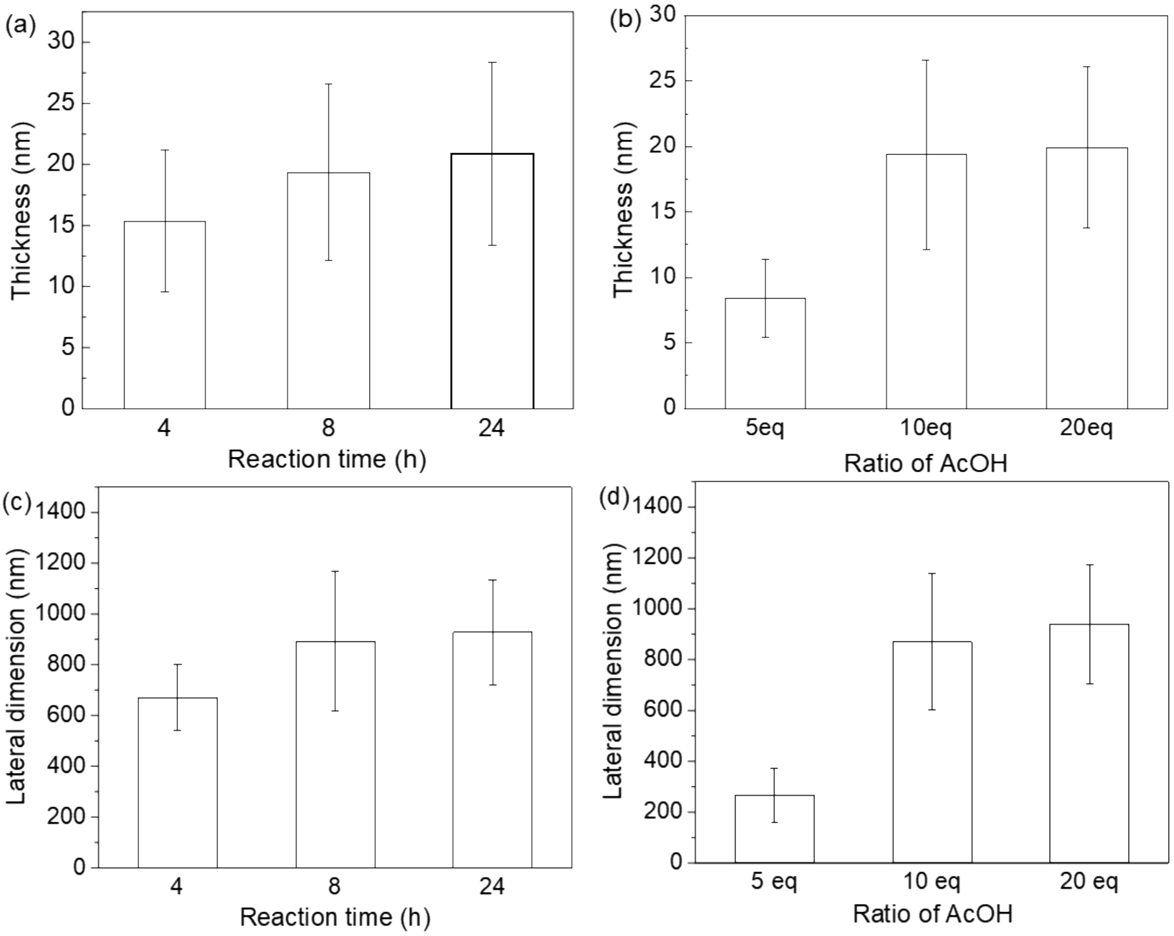


**Figure S3**. (a),(c) The effect of reaction time and, (b),(d) the effect of concentration of acetic acid modulator on the thickness of the nanosheet and their lateral dimension.

### 5. TEM image of *ns*-kgm$^{t\text{-Bu}}$

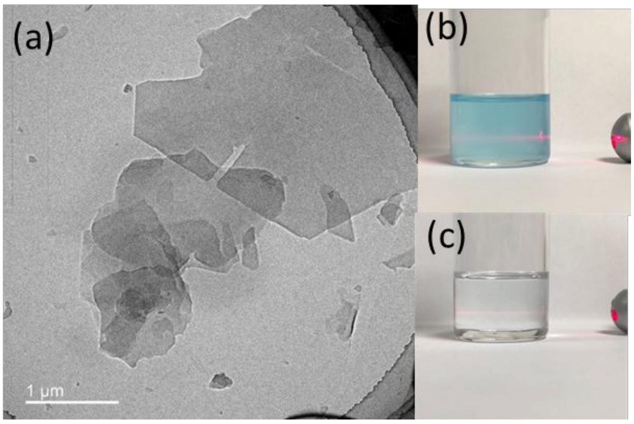


**Figure S4.** (a) TEM image of the *ns*-**kgm**$^{t\text{-Bu}}$ deposited on a carbon film; (b) the Tyndall effect of the suspension of nanosheets (b) in $CHCl_3$ and (c) after sonication

## 6. Pore size distribution analysis

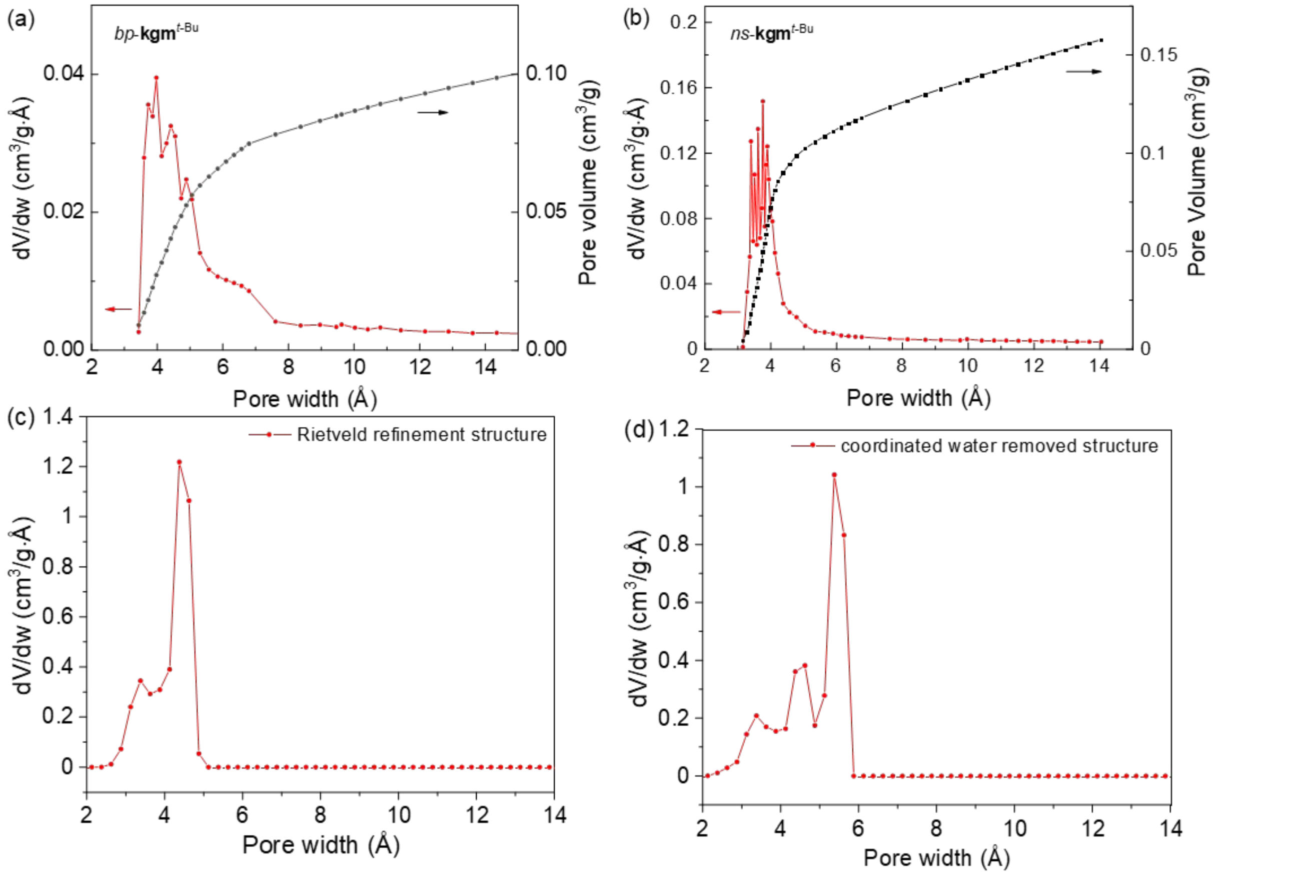


**Figure S5**. Pore size distribution (PSD) and cumulative pore volume plots based on Horvath-Kawazoe method from $N_2$ adsorption isotherms measured at 77 K of samples activated at 333K. (a) *bp*-**kgm**$^{t\text{-Bu}}$, (b) *ns*-**kgm**$^{t\text{-Bu}}$. (c) Calculated PSD on solvent-free structure from Rietveld refinement and (d) calculated PSD after removal of the axial water on the $[Cu_2(OOCR)_4]$ paddlewheel.

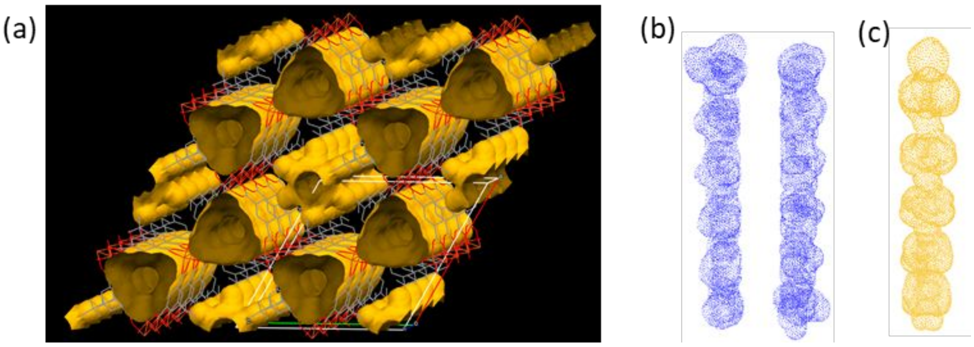


**Figure S6.** Illustration of channel shapes in **kgm**$^{t\text{-Bu}}$. Views of (a) the channel network, (b) the shape of channel I, and (c) the shape of channel II

## 7. Gravimetric adsorption isotherms and IAST analysis

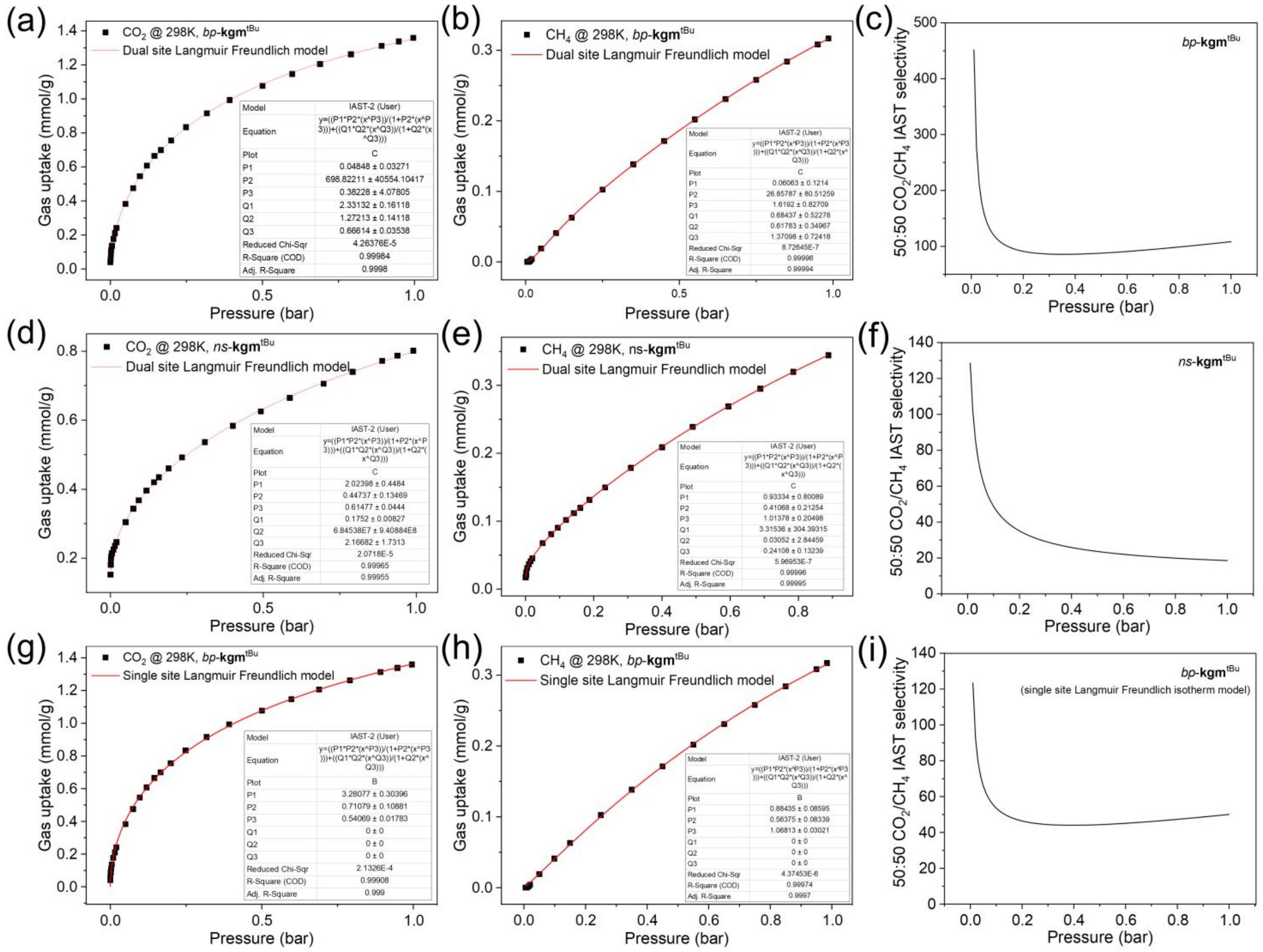


**Figure S7.** IAST fittings of adsorption isotherms at 298K for $CO_2$ and $CH_4$ in (a-b) *bp*-**kgm**$^{t\text{-Bu}}$ and (d-e) *ns*-**kgm**$^{t\text{-Bu}}$ using the dual site Langmuir Freundlich (DSLF) isotherm model and in (g-h) *bp*-**kgm**$^{t\text{-Bu}}$ using single site Langmuir Freundlich isotherm model (SSLF). IAST selectivity for a 50:50 mixture of $CO_2/CH_4$ based on fitted parameters for (c) *bp*-**kgm**$^{t\text{-Bu}}$, (f) *ns*-**kgm**$^{t\text{-Bu}}$ using the DSLF model and for (i) *bp*-**kgm**$^{t\text{-Bu}}$ using the SSLF model. The fitting of $CO_2$ isotherm of *ns*-**kgm**$^{t\text{-Bu}}$ using the SSLF model does not converge, which may be due to the high heterogeneity of the nanosheet morphology.

## 8. Thermogravimetric analysis

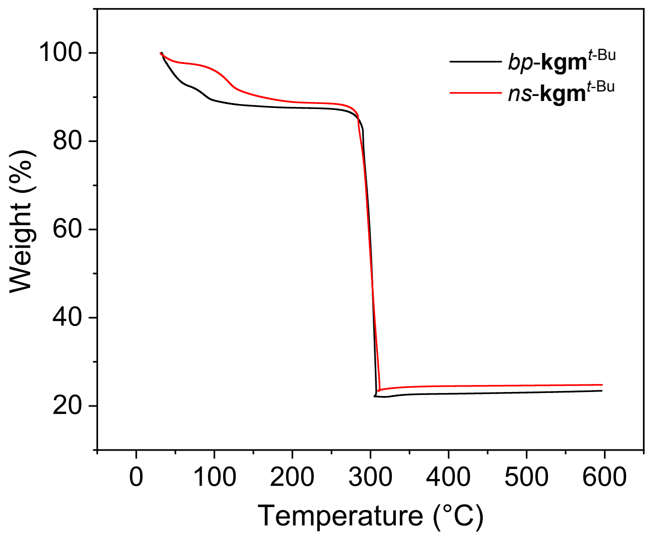


**Figure S8.** Thermogravimetric curves of *ns*-**kgm**$^{t\text{-Bu}}$ and *bp*-**kgm**$^{t\text{-Bu}}$ after solvent exchange with acetone.

## 9. Molecular dynamic simulations

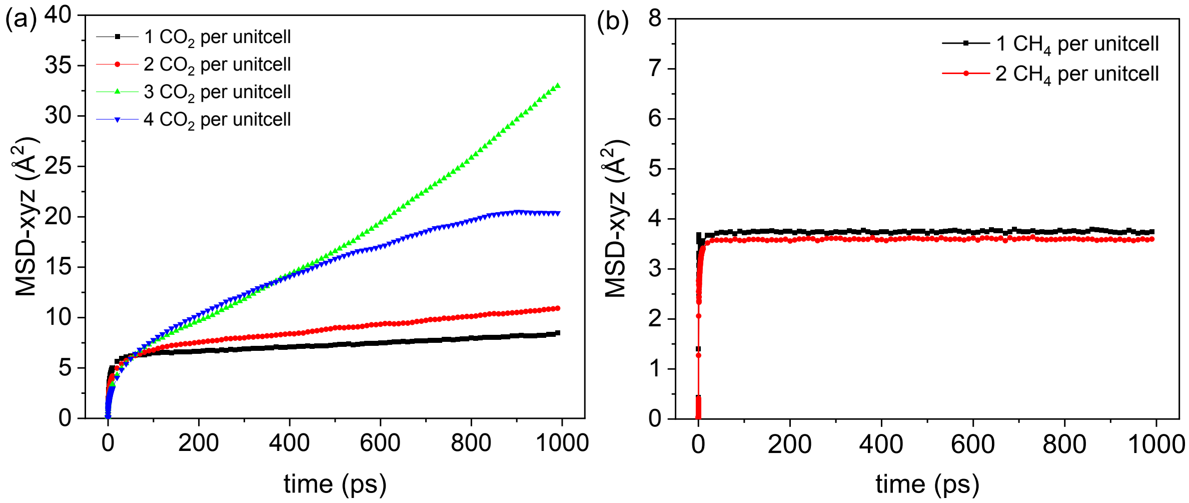


**Figure S9.** Mean squared displacement (MSD) as a function of time of (a) $CO_2$ and (b) $CH_4$ from the MD simulation. The enhancement of $CO_2/CH_4$ selectivity in *ns*-**kgm**$^{t\text{-Bu}}$ Matrimid membranes can be explained from the size-screening effect of the filler. The diffusion of $CH_4$ in the layered **kgm**$^{t\text{-Bu}}$ is hindered as the mean square displacement stagnated very quickly from the beginning. In contrast, $CO_2$ molecules can diffuse relatively freely due to its linear shape and smaller kinetic diameter. The diffusion "speeds up" upon increasing the loading of $CO_2$. This is probably also due to the increased guest-guest intermolecular interaction. At 4 molecules per cell, which is higher than the $CO_2$ uptake at 273 K, the diffusion is confined due to significantly reduced void within the MOF framework.

**Table S4** Diffusion coefficients of $CO_2$ from MD simulation

| $CO_2$ | Slope*/ $m^2\ s^{-1}$ | Diffusion coefficient/ $m^2\ s^{-1}$ | Linear Fitting $R^2$ |
|---|---|---|---|
| 1 per cell | $2.15\times10^{-11}$ | $3.58\times10^{-12}$ | 0.994 |
| 2 per cell | $4.23\times10^{-11}$ | $7.05\times10^{-12}$ | 0.998 |
| 3 per cell | $2.94\times10^{-10}$ | $4.9\times10^{-11}$ | 0.991 |
| 4 per cell | Nonlinear | | |

*: the initial nonlinear part was truncated. This nonlinear part is due to the system not fully equilibrated based on the simulation setup. Diffusion coefficient for the linear part is calculated

## 10. Comparison of gas separation performance of MMMs

**Table S5**. Comparison of $CO_2/CH_4$ separation performance of reported nanosheets Matrimid MMMs

| MOFs | Polymer | loading/wt% | Measurement Conditions | Feed | $P_{CO2}$ /Barrer | $\alpha_{CO2/CH4}$ | Ref. |
|---|---|---|---|---|---|---|---|
| CuBDC[a] | Matrimid 5218 | 8.2 | 3bar, 25 °C | 50/50, v/v | 4.1 | 78.7 | 9 |
| CuBDC | Matrimid 5218 | 12 | 5bar, 35 °C | 50/50, v/v | 6.3 | 38.0 | 10 |
| $NH_2$-MIL-53(Al)[b] | Matrimid 5218 | 8 | 3bar, 25 °C | 50/50, v/v | 13.3 | 31.1 | 11 |
| $NH_2$-MIL-53(Al) | Matrimid 5218 | 16 | 3bar, 25 °C | 50/50, v/v | 13.5 | 34.4 | |
| - | Matrimid 5218 | - | 3bar, 25 °C | Single gas | 7.1 | 53.5 | This work |
| *ns*-**kgm**$^{t\text{-Bu}}$ | Matrimid 5218 | 8 | 3bar, 25 °C | Single gas | 7.3 | 65.2 | |
| | Matrimid 5218 | 10 | 3bar, 25 °C | Single gas | 6.7 | 70.5 | |
| *bp*-**kgm**$^{t\text{-Bu}}$ | Matrimid 5218 | 8 | 3bar, 25 °C | Single gas | 6.5 | 57.4 | |
| | Matrimid 5218 | 10 | 3bar, 25 °C | Single gas | 6.0 | 59.1 | |

[a]CuBDC nanosheets, [b]$NH_2$-MIL-53(Al) nanosheets.

**Table S6.** Comparison of $CO_2/CH_4$ separation performance with reported nanosheet/polymer MMMs

| MOFs | Polymer | loading/wt% | Measurement Conditions | Feed | $P_{CO2}$ /Barrer | $\alpha_{CO2/CH4}$ | Ref. |
|---|---|---|---|---|---|---|---|
| Cu-BDC | 6FDA-DAM | 4 | 1bar, 25 °C | 50/50, v/v | 430.0 | 43.0 | 12 |
| Cu-BDC | PIM-1 | 4 | 1bar, 25 °C | 50/50, v/v | 2300 | 22 | |
| AlFFIVE-1-Ni[c] | 6FDA-DAT | 60.3 | 10bar, 35 °C | 10/90, v/v | 129.8 | 141.8 | 13 |
| AlFFIVE-1-Ni | 6FDA-DAM | 58.9 | 10bar, 35 °C | 10/90, v/v | 1095.4 | 41.2 | |
| AlFFIVE-1-Ni | 6FDA-DAM-DAT | 59.6 | 10bar, 35 °C | 10/90, v/v | 435.9 | 84.7 | |
| - | Matrimid 5218 | - | 3bar, 25 °C | Single gas | 7.1 | 53.5 | This work |
| *ns*-**kgm**$^{t\text{-Bu}}$ | Matrimid 5218 | 8 | 3bar, 25 °C | Single gas | 7.3 | 65.2 | |
| | Matrimid 5218 | 10 | 3bar, 25 °C | Single gas | 6.7 | 70.5 | |
| *bp*-**kgm**$^{t\text{-Bu}}$ | Matrimid 5218 | 8 | 3bar, 25 °C | Single gas | 6.5 | 57.4 | |
| | Matrimid 5218 | 10 | 3bar, 25 °C | Single gas | 6.0 | 59.1 | |

[c](001)-AlFFIVE-1-Ni nanosheets

## 11. PXRD of *ns*-kgm$^{t\text{-Bu}}$ Matrimid MMMs

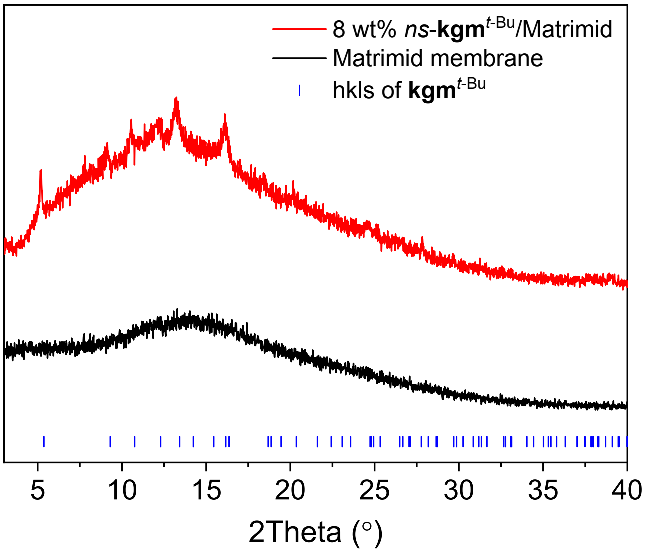


**Figure S10.** PXRD patterns of 8 wt% *ns*-**kgm**$^{t\text{-Bu}}$ Matrimid membrane and of pure Matrimid membrane with Bragg peak positions of **kgm**$^{t\text{-Bu}}$ shown in ticks.

## 12. Images of *ns*-kgm$^{t\text{-Bu}}$ Matrimid MMMs

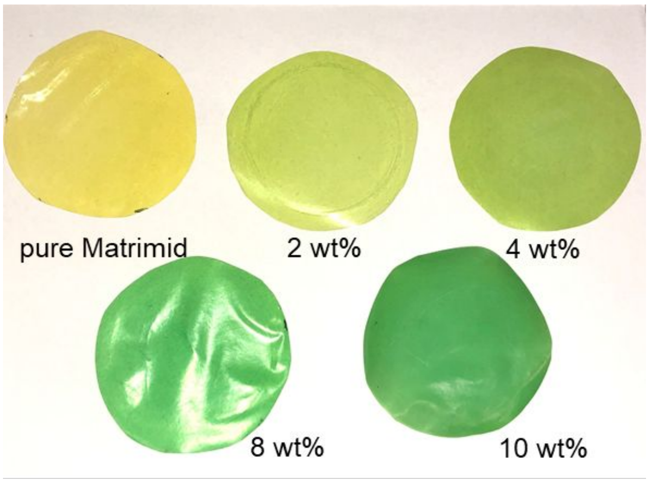


**Figure S11.** Images of the mixed-matrix membranes with different mass loading of *ns*-**kgm**$^{t\text{-Bu}}$ after gas permeation measurements.

## References


1 D.-X. Xue, Y.-Y. Lin, X.-N. Cheng and X.-M. Chen, *Cryst. Growth Des.*, 2007, **7**, 1332-1336.

2 O. V. Dolomanov, L. J. Bourhis, R. J. Gildea, J. A. Howard and H. Puschmann, *J. Appl. Crystallogr.*, 2009, **42**, 339-341.

3 Fitch, A.; Dejoie, C.; Covacci, E.; Confalonieri, G.; Grendal, O.; Claustre, L.; Guillou, P.; Kieffer, J.; de Nolf, W.; Petitdemange, S.; Ruat, M.; Watier, Y. ID22 – the High-Resolution Powder-Diffraction Beamline at ESRF. *J. Synchrotron Radiat.* **2023**, *30* (5), 1003–1012.

4 L. N. McHugh, L. J. Olivera Perez, P. S. Wheatley, D. B. Cordes, A. M. Z. Slawin and R. E. Morris, *CrystEngComm*, 2019, **21**, 5387-5391.

5 P. Giannozzi, S. Baroni, N. Bonini, M. Calandra, R. Car, C. Cavazzoni, D. Ceresoli, G. L. Chiarotti, M. Cococcioni, I. Dabo, A. Dal Corso, S. de Gironcoli, S. Fabris, G. Fratesi, R. Gebauer, U. Gerstmann, C. Gougoussis, A. Kokalj, M. Lazzeri, L. Martin-Samos, N. Marzari, F. Mauri, R. Mazzarello, S. Paolini, A. Pasquarello, L. Paulatto, C. Sbraccia, S. Scandolo, G. Sclauzero, A. P. Seitsonen, A. Smogunov, P. Umari and R. M. Wentzcovitch, *J. Phys. Condens. Matter.*, 2009, **21**, 395502.

6 P. Giannozzi, O. Andreussi, T. Brumme, O. Bunau, M. Buongiorno Nardelli, M. Calandra, R. Car, C. Cavazzoni, D. Ceresoli, M. Cococcioni, N. Colonna, I. Carnimeo, A. Dal Corso, S. de Gironcoli, P. Delugas, R. A. DiStasio, A. Ferretti, A. Floris, G. Fratesi, G. Fugallo, R. Gebauer, U. Gerstmann, F. Giustino, T. Gorni, J. Jia, M. Kawamura, H. Y. Ko, A. Kokalj, E. Kucukbenli, M. Lazzeri, M. Marsili, N. Marzari, F. Mauri, N. L. Nguyen, H. V. Nguyen, A. Otero-de-la-Roza, L. Paulatto, S. Ponce, D. Rocca, R. Sabatini, B. Santra, M. Schlipf, A. P. Seitsonen, A. Smogunov, I. Timrov, T. Thonhauser, P. Umari, N. Vast, X. Wu and S. Baroni, *J. Phys. Condens. Matter.*, 2017, **29**, 465901.

7 D. Dubbeldam, S. Calero, D. E. Ellis and R. Q. Snurr, *Mol. Simul.* , 2016, **42**, 81-101.

8 D. Dubbeldam and R. Q. Snurr, *Mol. Simul.*, 2007, **33**, 305-325.

9 T. Rodenas, I. Luz, G. Prieto, B. Seoane, H. Miro, A. Corma, F. Kapteijn, I. X. F. X. Llabres and J. Gascon, *Nat. Mater.*, 2015, **14**, 48-55.

10 M. Shete, P. Kumar, J. E. Bachman, X. Ma, Z. P. Smith, W. Xu, K. A. Mkhoyan, J. R. Long and M. Tsapatsis, *J. Membr. Sci.*, 2018, **549**, 312-320.

11 A. Pustovarenko, M. G. Goesten, S. Sachdeva, M. Shan, Z. Amghouz, Y. Belmabkhout, A. Dikhtiarenko, T. Rodenas, D. Keskin, I. K. Voets, B. M. Weckhuysen, M. Eddaoudi, L. de Smet, E. J. R. Sudholter, F. Kapteijn, B. Seoane and J. Gascon, *Adv. Mater.*, 2018, **30**, e1707234.

12 Y. Yang, K. Goh, R. Wang and T.-H. Bae, *Chemical Communications*, 2017, **53**, 4254-4257.

13 S. J. Datta, A. Mayoral, N. M. S. Bettahalli, P. M. Bhatt, M. Karunakaran, I. D. Carja, D. Fan, P. G. M. Mileo, R. Semino, G. Maurin, O. Terasaki and M. Eddaoudi, *Science*, 2022, **376**, 1080-1087.
